# MOF Coating Enhances the Ion Tolerance of Micromotors

**DOI:** 10.1002/anie.202508001

**Published:** 2025-06-03

**Authors:** Leyan Ou, Kunfeng Liu, Yifan Zhang, Wanyuan Li, Zixian Liang, Dapeng Lei, Hao Sun, Mojun Chen, Jizhuang Wang, Jinyao Tang, Dan Li

**Affiliations:** ^1^ College of Chemistry and Materials Science, Guangdong Provincial Key Laboratory of Supramolecular Coordination Chemistry Jinan University Guangzhou Guangdong 510632 P.R. China; ^2^ Department of Chemistry The University of Hong Kong Pokfulam Road Hong Kong P.R. China; ^3^ Guangdong Provincial Key Laboratory of Spine and Spinal Cord Reconstruction, The Fifth Affiliated Hospital (Heyuan Shenhe People's Hospital) Jinan University Heyuan 517000 P.R. China; ^4^ Smart Manufacturing Thrust, Systems Hub The Hong Kong University of Science and Technology (Guangzhou) Guangzhou 511458 P.R. China

**Keywords:** Ion tolerance, Light‐driven micromotor, MOF coating, NIR‐driven, Silicon micromotor

## Abstract

Electrophoretic‐driven micro/nanomotors (EMNMs) offer great potential for biomedical applications due to their design flexibility. However, they face challenges in high‐salt environments, where ionic quenching disrupts propulsion by collapsing the electrical double layer. This study introduces a versatile strategy by coating EMNMs with a MOF porous scaffold (ZIF‐8), which acts as ion‐conductive channels that replace the electrical Debye layers and support propulsion in high‐salt solutions. Through a heteroepitaxial growth process, ZIF‐8 was precisely coated on silicon micromotors, a typical model for EMNMs, significantly enhancing their ion tolerance. By optimizing both the MOF layer and the geometry factor, the micromotors achieved effective motion in PBS solution, comparable to blood salt levels, with their ion tolerance (*EI*_50_) improving by up to 266 times compared to uncoated micromotors. Additionally, the micromotors maintained stable, controllable motion under 980 nm NIR light, even when passing through an artificial blood vessel covered with biological tissues. In addition, the ZIF‐8 coating offers drug‐loading capabilities and pH‐responsive release, along with biocompatibility, making these micromotors suitable for targeted drug delivery. This MOF coating strategy is versatile and scalable, and can be extended to other types of EMNMs, significantly enhancing their ion tolerance and unlocking new possibilities for biomedical applications.

## Graphical abstract

This work reported a novel method to significantly enhance the ion tolerance of EMNMs using a MOF coating via controllable heteroepitaxial growth, enabling effective motion in high‐salt environments and propulsion through biological tissues under 980 nm NIR light, with potential applications in targeted drug delivery.

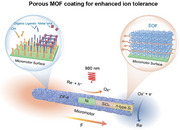

## Introduction

Micro/nanomotors (MNMs) are capable of converting various types of energy—such as chemical, magnetic, acoustic, and light energy—into mechanical motion, enabling them to perform complex tasks at the micro‐ and nanoscale level.^[^ ^1^, ^2^, ^3^, ^4^, ^5^, ^6^, ^7^ ^]^ Equipped with distinctive advantages, including precise controllability, efficient propulsion, and facile functionalization, MNMs present tremendous potential for biomedical applications.^[^ ^8^, ^9^, ^10^, ^11^, ^12^, ^13^ ^]^ Notable examples include enzyme‐powered MNMs for targeted drug delivery,^[^ ^14^, ^15^, ^16^ ^]^ bubble‐propelled micromotors for minimally invasive surgery,^[^ ^17^, ^18^, ^19^, ^20^ ^]^ and magnetically guided MNMs for precision therapy.^[^ ^21^, ^22^, ^23^, ^24^ ^]^ Among the various types of MNMs, electrophoretic‐driven MNMs (EMNMs), including electrophoresis, self‐electrophoresis, and electrolyte diffusiophoresis, have garnered significant interest.^[^ ^25^, ^26^, ^27^, ^28^, ^29^, ^30^, ^31^, ^32^ ^]^ These EMNMs rely on asymmetric chemical reactions on their surfaces to generate propulsion, which can be easily modulated and offers substantial design flexibility.^[^ ^33^, ^34^ ^]^ Such EMNMs are expected to serve as ideal artificial systems for mimicking biological chemotaxis—cell migration guided by chemical gradients—providing a promising platform for targeted drug delivery and precision medicine.^[^ ^35^, ^36^ ^]^

However, EMNMs face a substantial obstacle in high‐concentration electrolyte environments, such as blood and other biological fluids.^[^ ^37^ ^]^ This ionic quenching behavior is well explained by the classical Helmholtz–Smoluchowski electrokinetic theory, which predicts that high ionic strength collapses the Debye layer, completely suppressing electric‐field‐driven propulsion. Consequently, overcoming these salt concentrations, often exceeding 150 mM in biological media, is essential for biomedical applications. To evaluate the extent of this limitation and compare the ion tolerance performance among different MNMs systems, a quantitative metric is required. The ion tolerance of MNMs, defined as the motion ability of micromotors in ionic solution, can be characterized by the “Median Effective Ionic Strength” (*EI*₅₀), representing the salt concentration at which MNMs’ speed is reduced to half of its initial speed value. For most conventional EMNMs, the *EI*₅₀ is typically below 0.1 mM, underscoring the challenge of achieving effective propulsion in high‐salt environments. Modifications to the classical theory have highlighted the role of surface conductivity,^[^ ^33^, ^34^ ^]^ quantified by the Dukhin number (*Du*), defined as: $Du=\frac{K^{\sigma }}{aK^{L}}\;$, where $“K^{\sigma }”$ is the surface conductivity, $“K^{L}”$ is the solution conductivity and “a” is the particle's geometry factor. Previous studies have shown that increasing surface conductivity with a polyelectrolyte layer and optimizing geometry can enhance ion tolerance.^[^ ^38^ ^]^ Moreover, various designs, such as porous MNMs, ultra‐small nanomotors, and surface‐modified structures, have demonstrated improved ion tolerance.^[^ ^8^, ^39^, ^40^, ^41^, ^42^, ^43^, ^44^, ^45^, ^46^, ^47^, ^48^, ^49^, ^50^, ^51^, ^52^, ^53^, ^54^, ^55^ ^]^ However, a general strategy to overcome ionic quenching for EMNMs remains elusive. Although the precise mechanism is still under investigation, porous MNMs have exhibited high ion tolerance,^[^ ^39^, ^40^ ^]^ suggesting that a facile coating of porous structures could provide a general solution.

**Figure 1:**
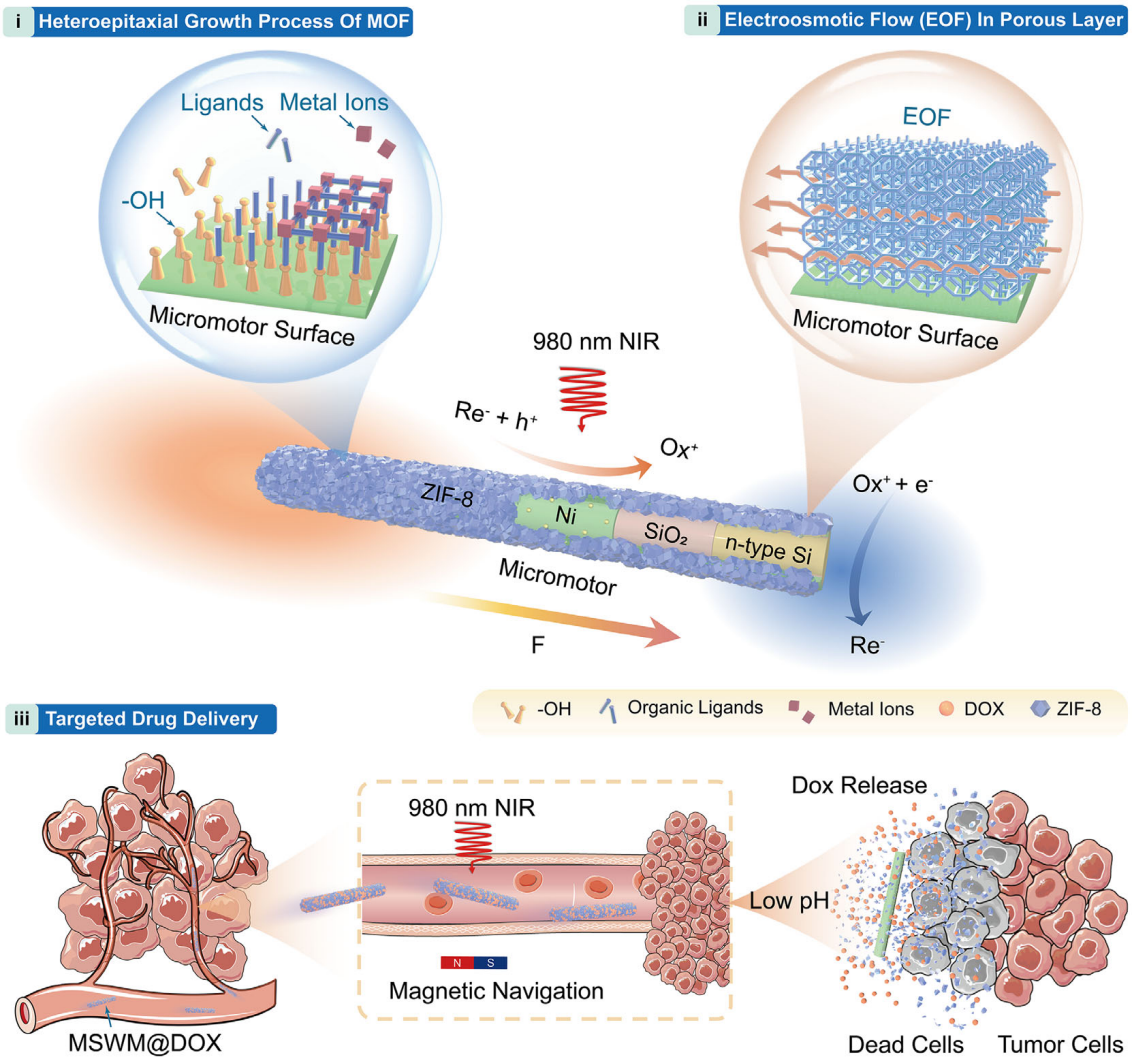
Schematic illustration of the design and operation of MOF‐coated silicon wire micromotors (MSWMs). The metal–insulator–semiconductor (MIS) structure was constructed by integrating n‐type silicon nanowires with an insulating SiO_2_ layer and Ni metal layer. Gold nanoparticles were subsequently introduced, functioning as electrocatalysts. A dense ZIF‐8 film was coated onto the surface, forming the MSWMs, which operate as a surface conductive “Debye layers” that support the electroosmosis flow. Upon exposure to 980 nm near‐infrared (NIR) light, the micromotors generate a photovoltage, initiating electrochemical reactions. Ferrocene‐based reversible shuttles—specifically ferrocenemethanol (MFc) and its cationic form (MFc⁺)—serve as the fuel, shuttling between the photoanode and photocathode. The asymmetric distribution of charged products during the redox cycle generates an electric field across the electrodes, driving the micromotor forward. Additionally, the ZIF‐8 coating enables efficient drug loading and pH‐responsive release, while the incorporation of magnetic components enhances controllability, making the MSWMs highly suitable for targeted drug delivery. This multifunctional design provides a robust strategy to address ion tolerance challenges for electrophoretic‐driven MNMs (EMNMs), broadening their potential for biomedical applications.

As metal–organic framework (MOF) has been extensively studied as an ion conductor,^[^ ^56^, ^57^, ^58^, ^59^ ^]^ it is feasible to exploit the highly ordered pores as electrokinetic layers to support fluidic pumping in a high‐salt environment. In this study, we selected zeolitic imidazolate framework‐8 (ZIF‐8), a well‐studied MOF, as a porous coating for silicon‐based micromotors. Through a heteroepitaxial growth process,^[^ ^60^ ^]^ we successfully synthesized dense ZIF‐8 coatings on silicon micromotors, a typical light‐driven self‐electrophoresis micromotor as the model, significantly enhancing their ion tolerance in high‐salt solutions (Figure 1). The proposed quantitative model supports the observed improvement in ion tolerance behavior. The propulsion mechanism of the micromotor is based on light‐induced self‐electrophoresis. Upon NIR illumination, photoexcited charge carriers are separated within the metal–insulator–semiconductor (MIS) junction. This spatial separation induces a photovoltage that drives asymmetric redox reactions at the two ends of the micromotor. The resulting asymmetric ionic distribution generates a localized electric field that propels the charged micromotor via a self‐electrophoretic mechanism. Furthermore, the silicon micromotors exhibited efficient propulsion under 980 nm near‐infrared (NIR) light—a wavelength known for deep tissue penetration with minimal damage—and demonstrated precise magnetic controllability, which is advantageous for biomedical applications, such as targeted drug delivery. Their extendibility to other EMNM systems, along with efficient drug‐loading capabilities, makes them promising candidates for biomedical applications, particularly in targeted drug delivery. Therefore, this work aims to develop a simple and generalizable surface modification strategy based on MOF porous coatings to enhance the ionic tolerance of EMNMs, and to elucidate the propulsion mechanism in high ionic strength environments.

## Results and Discussion

### Fabrication and Characterization of MSWMs

**Figure 2:**
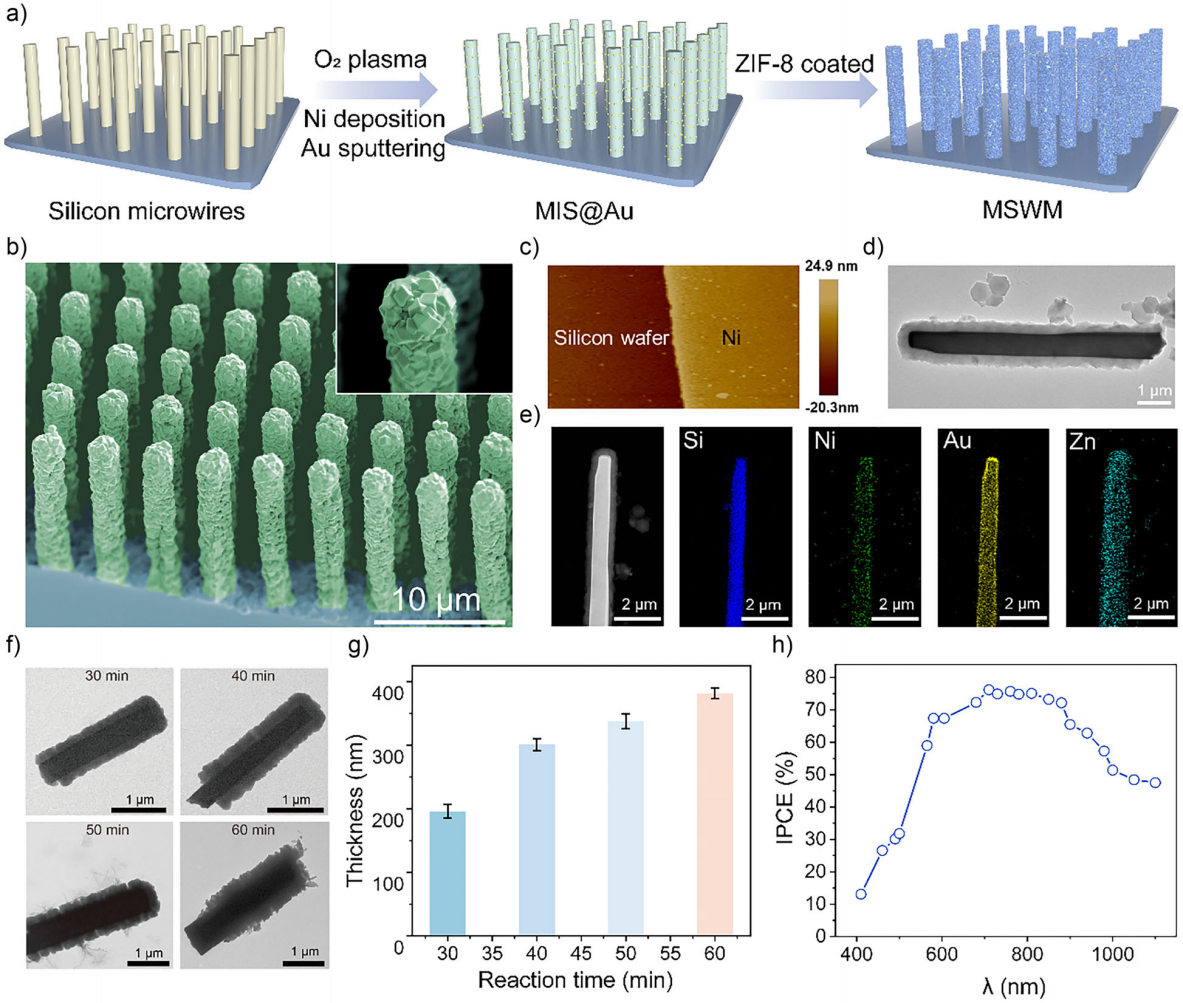
Synthesis process and micromotor characterization. a) Schematic illustration of the MSWMs synthesis process. b) Scanning Electron Microscopy (SEM) image of the MSWMs array on a silicon substrate, with an enlarged view shown in the inset. c) Atomic Force Microscopy (AFM) image revealing the thickness of the Ni layer. d) Transmission Electron Microscopy (TEM) image of a typical MSWM. e) Energy Dispersive X‐ray (EDX) mapping displaying the elemental distribution of Si, Ni, Au, and Zn on a typical MSWM. f) TEM images of the ZIF‐8 film thickness at different growth times: 30, 40, 50, and 60 min. g) Plot depicting the film thickness as a function of reaction time. Error bars represent the SD of the averaged values from three measurements. h) Incident photon‐to‐current efficiency (IPCE) spectra of MSWMs across wavelengths ranging from 400 to 1100 nm.

In this study, our previously reported MIS silicon wire micromotor was utilized as a model system due to its modulable light‐responsive migration behavior and well‐characterized self‐electrophoresis mechanism.^[^ ^61^ ^]^ As the porous coating, we selected ZIF‐8, a widely studied MOF known for its high porosity, tunable chemical properties, biocompatibility, and ease of synthesis.^[^ ^62^ ^]^ As illustrated in Figure 2a, ZIF‐8 was deposited onto the silicon micromotor surface using a heteroepitaxial growth process, enabling precise control over the coating thickness.

The fabrication process of the MOF‐coated silicon wire micromotors (MSWMs) is outlined in Figure 2a. Large‐scale silicon wire arrays were prepared via photolithography and wet‐etching methods. Specifically, dot arrays were patterned on a silicon wafer by photolithography, followed by wet etching to produce silicon wires of adjustable lengths, while the diameter was controlled by tuning dot sizes and oxidation/etching process. Followingly, the insulator layer was created by oxygen plasma oxidation, forming a thin SiO₂ film, and subsequently, thermal deposition of a Ni layer to complete the MIS structure. Gold nanoparticles were introduced as electrocatalysts, resulting in the formation of MIS@Au micromotors.

For the heteroepitaxial growth process of the ZIF‐8 coating, the as‐prepared MIS@Au micromotor array was first immersed in the 2‐methylimidazole solution. Subsequently, the zinc precursor solution was introduced into the 2‐methylimidazole solution. The reaction was carried out at room temperature and atmospheric pressure, with varying reaction times to control the coating thickness. Following the drying process, the micromotor assembly yielded MSWMs (further fabrication details are available in the Supporting Information). As shown in Figure 2b, scanning electron microscopy (SEM) revealed that the micromotors exhibited an average length of ∼15 µm. The oxygen plasma oxidation method was applied to create a SiO_2_ layer of approximately 2.7 nm thickness, as depicted in Figure S1. A Ni layer of ∼25 nm thickness (Figure S2), deposited via thermal evaporation, constitutes the metal component of the MIS junction (Figure 2c). The ferromagnetic properties of the MSWMs are validated by the hysteresis loop, as shown in Figure S3. Transmission electron microscopy (TEM) images (Figure 2d) confirmed the uniform ZIF‐8 coating on the MIS@Au micromotors, validating the successful functionalization. Elemental analysis further corroborated the structural integrity of the MSWM, demonstrating the presence of Ni, Au, and Zn uniformly distributed on the surface (Figure 2e). The zeta potential of MSWMs was measured to be positive (Figure S4).

The growth kinetics of the ZIF‐8 coating are depicted in Figure 2f, showing thickness profiles at different reaction times (30–60 min). As summarized in Figure 2g, the coating thickness exhibits a near‐linear dependence on the reaction time, facilitating precise control over the layer dimensions. The porous ZIF‐8 coating acts as a surface scaffold, maintaining surface conductive “Debye layers” that support ion flow and prevent the quenching of self‐electrophoresis. However, excessively thick coatings hinder fuel diffusion, underscoring the importance of optimizing the coating thickness to balance propulsion efficiency and ion tolerance.

Although light‐responsive micromotors offer promise for biological applications, challenges remain, including limited ion tolerance in physiological environments and reliance on external light sources. Conventional light‐driven micromotors often use UV light, which is incompatible with biological tissues. Although visible light offers improved biocompatibility, it suffers from limited tissue penetration. NIR light offers deeper penetration and enhanced biocompatibility, making it ideal for biomedical applications. Silicon‐based junctions exhibit broad‐spectrum light absorption, making them suitable candidates for NIR‐driven propulsion. To evaluate the performance of the silicon micromotor under NIR light, we measured the incident photon‐to‐current efficiency (IPCE) across various wavelengths. As shown in Figure 2h, the micromotors exhibit high photoelectrochemical efficiency, with robust photon‐to‐current conversion even in the NIR region. This indicates that these micromotors are well‐suited for future biological applications, where deep tissue penetration is essential for remote activation and navigation. The inherent ability of NIR light to reach subcutaneous and vascular regions makes it a practical and effective stimulus in biological settings, particularly in scenarios where visible light cannot reach the target area. These results highlight the potential of MSWMs for NIR‐driven applications, providing a promising strategy for integrating light‐responsive micromotors in biological environments.

### Ion Tolerance Enhancement With MOF Coating

**Figure 3:**
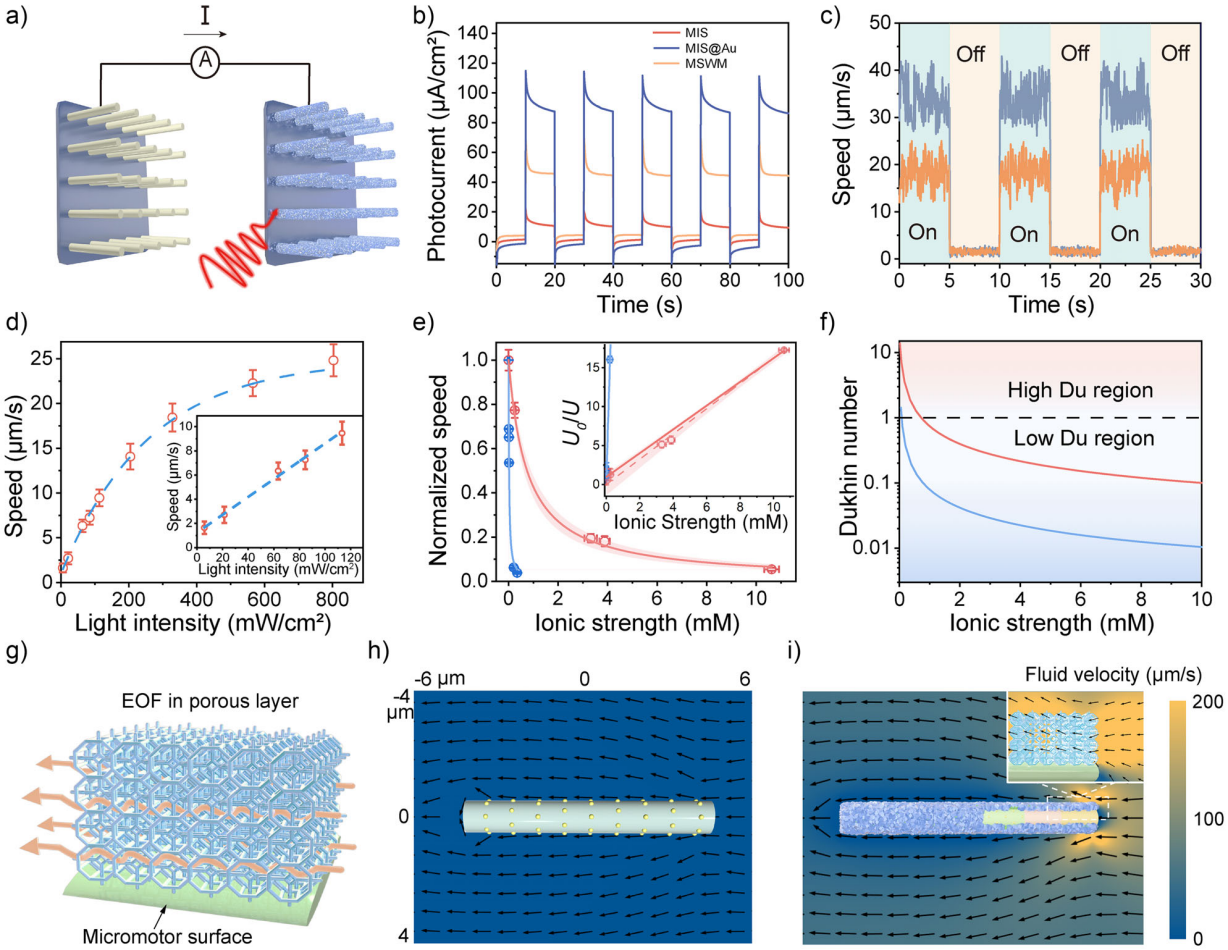
Motion analysis and enhanced ion tolerance of MSWMs. a) Schematic illustration of the photocurrent measurement setup for MSWMs. b) Short‐circuit current responses of the MIS structure (red), MIS@Au micromotor (blue), and MSWM (orange) immersed in MFc solution under chopped 980 nm NIR illumination (≈1.2 W cm^−2^). c) Comparison of the migration speed of MSWMs (orange) and MIS@Au micromotors (blue) under a chopped 980 nm NIR light (750 mW cm^−2^). d) Dependence of MSWM migration speed on 980 nm NIR light intensity. The inset highlights the quasi‐linear relationship at low light intensities (0–120 mW cm^−2^). Error bars represent the SD of the averaged values from three measurements. e) Normalized migration speeds of MIS@Au micromotor (blue) and MSWM (red) as a function of ionic strength, shown with experimental data (sphere markers) and theoretical curve (solid lines). Shaded bands indicate the 95% confidence interval. The inset displays the linear relationship between the normalized inverse speed and ionic strength, following Equation 1. The red solid line represents model predictions, while the dashed line and shaded regions show the fitted curves and 95% confidence interval. Error bars represent the SD of the averaged values from three measurements. f) Dukhin number comparison of MIS@Au micromotor (blue) and MSWM (red). g) Schematic illustration of the EOF in a porous layer. h) Numerical simulation of the flow field (black arrows) and flow velocity (color map) of bare MIS@Au micromotor. i) Numerical simulation of the flow field (black arrows) and flow velocity (color map) of MSWM.

The propulsion of the silicon micromotor originates from a photoelectrochemical reaction‐driven self‐electrophoresis. Upon exposure to 980 nm NIR light, a photovoltage is generated across the MIS junction, initiating electrochemical redox reactions. This leads to localized ion generation, creating an asymmetric charge gradient and an electric field around the micromotor. The electric field interacts with the electrical double layer (EDL), driving electroosmotic flow (EOF) and propelling the micromotor forward. The current flow generated by the photoelectrochemical reaction was confirmed through direct photocurrent measurements (Figure 3a). As shown in Figure 3b, the photocurrent was measured with and without gold nanoparticles as electrocatalysts under modulated light conditions. The results confirm that the presence of gold nanoparticles significantly enhances the electrochemical reactions. However, the ZIF‐8 coating on the micromotor partially hinders mass transfer, slightly reducing the photocurrent of MSWMs compared to uncoated micromotors.

To assess the propulsion performance, we examined the micromotor migration under 980 nm NIR illumination, employing an instantaneous “ON/OFF” light pattern (see Movie S1). During the “ON” state, the micromotor moves directionally, while the “OFF” state results in random Brownian motion. As shown in Figure 3c, the motion speed under alternating illumination was quantified. The ZIF‐8 coating causes a slight reduction in migration speed compared to the uncoated MIS@Au micromotor, likely due to reduced fuel diffusion caused by the dense ZIF‐8 layer. Propulsion speed (*U*) relates to the effective current (*J*) and inversely to the diffusion coefficient (*D_ion_*) of ionic products, following $U\propto \frac{J}{D_{ion}}$.^[^ ^28^ ^]^ Unlike conventional H⁺‐based propulsion fuel sources like H₂O₂, glucose, and hydroquinone/benzoquinone, this study employs MFc⁺ ions, which have a much lower diffusion coefficient (approximately 1/37 of H⁺). As shown in Figure 3d, MFc⁺ ions allow for rapid propulsion saturation at lower current densities, thereby enhancing propulsion efficiency. The use of highly reversible redox eliminates the need for highly reactive chemical fuels, minimizing toxicity concerns and enabling precise propulsion through light as the sole external energy input.

As previously stated, the propulsion of MSWM relies on self‐electrophoresis. However, in high‐salt environments, EMNMs tend to lose motility. This occurs due to the compression of the Debye layer from ∼100 to ∼1 nm,^[^ ^27^, ^63^ ^]^ caused by electric field shielding effects, as predicted by the Helmholtz–Smoluchowski theory. This compression quenches the electrophoretic motion, reducing the micromotor's propulsion efficiency. In contrast, the porous ZIF‐8 coating mitigates this effect by acting as a scaffold that maintains electrical “Debye layers” even in high‐salt solutions. The ZIF‐8 coating provides ion‐conductive channels that prevent the collapse of the Debye layer, preserving the self‐electrophoresis mechanism. Therefore, the porous coating enhances ion tolerance by supporting EOF. The fundamental origin of all electrokinetic phenomena lies in the ion flow through the interfacial EDL. The efficiency of such phenomena, including electrophoresis and electrolyte diffusiophoresis, is quantified by the *Du*, which reflects the ratio between surface conductivity *K* ^σ^ and bulk solution conductivity *K^L^*, expressed in Equation 1.

(1) $$Du=\frac{K^{\sigma }}{aK^{L}}\;$$

where *a* is the characteristic size of the particle. For uncoated micromotors, the surface conductivity becomes negligible compared to the bulk conductivity in the presence of external electrolytes, resulting in a low *Du* (*Du* ≪ 1).^[^ ^63^ ^]^ In contrast, the porous coating significantly increases surface conductivity by introducing a high specific surface area layer that adds surface charges to the micromotor. This enhancement ensures that the *Du* remains high, even in electrolyte‐rich environments, boosting the micromotor's ion tolerance and electrokinetic efficiency.

To quantify ion tolerance, the normalized reciprocal speed ratio (*U*₀/*U*) was plotted against ionic strength, following the equation:

(2) $$\frac{U_{0}}{U}=1+\frac{1}{E_{I50}}C$$

where *EI*_50_ is the critical ionic strength at which the speed is reduced by 50%. Substituting into the following relation:

(3) $$EI_{50}=\frac{K^{\sigma }}{a\wedge_{m}}\;$$

where ∧_*m*_ is the molar conductivity, we can determine the micromotor's surface conductivity. Using a custom in situ electrical conductivity measurement device, we measured the migration speed of micromotors in different ionic‐strength solutions and normalized the speeds to obtain the *EI*₅₀ value.

As shown in Figure 3e, the uncoated micromotor (blue line) lost motility rapidly as the NaCl concentration increased. Equation 2 is applied to plot the ionic strength measured dependence curve, and the *EI*₅₀ value was calculated to be 0.020 ± 0.001 mM. In contrast, the MSWM (orange line) maintained directional motion even in 10 mM NaCl solution, with an *EI*₅₀ value of 0.748 ± 0.042 mM, indicating enhanced ion tolerance. Surface conductivity (*K* ^σ^) was determined to be (5.58 ± 0.14) × 10^−8^ S · □. The experimental results, depicted in the inset of Figure 3e, further confirmed the expected linear relationship, with the ZIF‐8 coating resulting in a smaller slope, indicating improved ion tolerance.

To further analyze the significance of surface conductivity, we compared the *Du* of coated and uncoated micromotors with the same geometry (length: ∼7.5 µm, diameter: ∼1.9 µm). As shown in Figure 3f, uncoated micromotors exhibited *Du* ≪ 1, indicating that most ion flow occurred in the bulk solution away from the micromotor, leading to minimal EOF and reduced propulsion. In contrast, the porous ZIF‐8 coating provided sufficient ion permeability, ensuring that ion flow primarily occurred through the MOF layer. This generated EOF that contributed to propulsion, dramatically increasing the *Du* number and enhancing the micromotor's ion tolerance (Figure 3f). Furthermore, numerical simulation was applied to calculate the fluid flow around the micromotor. Figure 3g illustrates the schematic of the EOF within the ZIF‐8 layer. As expected, in NaCl solution, the EOF on the bare micromotor is almost completely suppressed by the ion‐shielded electrophoretic field (Figure 3h). In contrast, EOF was observed within the ZIF‐8 layer (Figure 3i), demonstrating that the micromotor possesses sufficient propulsion force, which is also the source of the enhanced ion tolerance.

### Enhanced Ion Tolerance With Geometry Optimization

**Figure 4:**
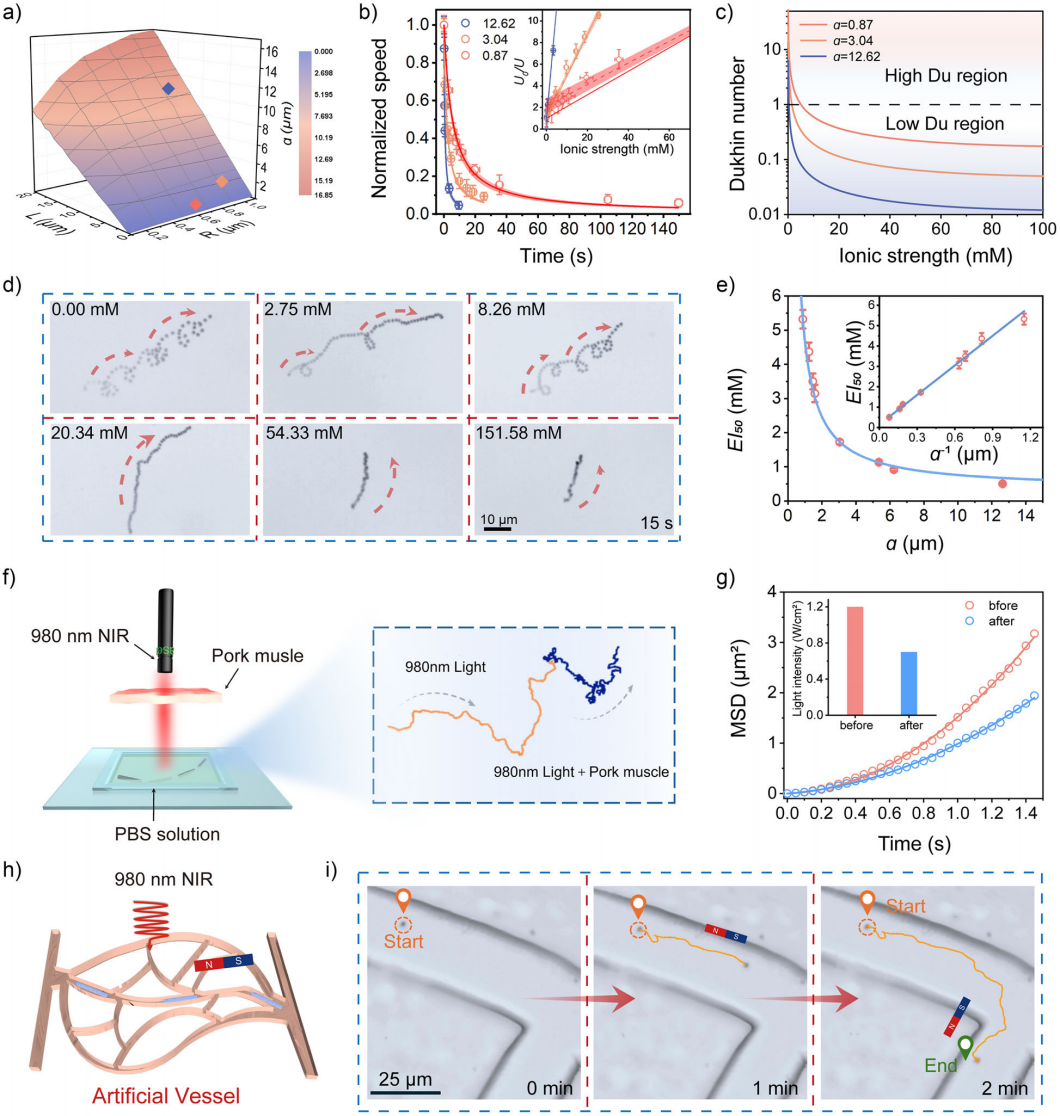
Geometry optimization for enhanced ion tolerance. a) The relationship between the geometry factor “a” and the micromotor's dimensions. b) The ionic strength dependence of the normalized speed for short (blue), medium (orange), and long (red) micromotors. The inset shows the linear relationship between the normalized reciprocal speed and ionic strength, with solid lines representing predictions from Equation 2. Dashed lines and the shaded bands indicate fitted plots with a 95% confidence interval. Error bars represent the SD of the averaged values from three measurements. c) The *Du* plotted as a function of ionic strength for micromotors with different geometries. d) The trajectories of a typical MSWM in different ionic strengths of the NaCl solution. e) The *EI*_50_ values plotted against the geometric factor “a”. Data points correspond to the values from Table S1. Error bars represent the SD of the averaged values from three measurements. f) The trajectory of a typical MSWM in PBS solution, shown before (orange line) and after (blue line) placing a 3 mm pork muscle tissue between the 980 nm NIR light and the micromotor. g) Mean Square Displacement (MSD) data for the MSWM driven by 980 nm NIR light. The inset shows the light intensity before and after penetrating the pork tissue. h) Schematic illustration of the MSWM's navigation process in an artificial vessel driven by 980 nm NIR light. i) The trajectories of MSWM in the artificial vessel.

Although the ZIF‐8 coating significantly enhances ion tolerance, the *EI*_50_ value remains insufficient for applications in high‐concentration electrolyte environments, such as biological fluids. To further improve ion tolerance, optimizing the geometry of the micromotors can be effective, as suggested by the relationship in the *Du* equation ($Du=\frac{K^{\sigma }}{aK^{L}}\;$). To explore the influence, we adjusted the geometry of the micromotors to enhance their ion tolerance. As shown in Figure 4a, numerical simulations reveal that the geometric factor “a” is primarily influenced by the micromotor length and has minimal dependence on its diameter. To systematically investigate this relationship, we controlled the etching parameters to obtain silicon wires of different lengths. Through multiple thermal oxidation processes, we achieved various diameters, followed by the coating of ZIF‐8 on the surface of the MIS@Au micromotor array, resulting in MSWM micromotors with different lengths and diameters, as shown in Figure S5. Subsequently, we measured the *EI*_50_ values of ZIF‐8‐coated micromotors with different diameters and lengths, correlating these values with their respective geometric factors “a”, as illustrated in Figure 4b. As the micromotor length decreased, the ion tolerance improved, reaching 150 mM, comparable to the salt concentration in blood. Specifically, the geometric factor “a” decreased from 12.62 µm (length: ∼15.1 µm, diameter: ∼1.1 µm) to 3.04 µm (length: ∼4.0 µm, diameter: ∼900 nm) and further to 0.87 µm (length: ∼2.0 µm, diameter: ∼700 nm). Correspondingly, their *Du* values increased, as shown in Figure 4c.

In Figure 4d, a time‐lapse sequence shows the motion of a micromotor under various NaCl concentrations (see Movie S2). As demonstrated in Figure 4e, the *EI*_50_ value is inversely proportional to the geometric factor “a”, consistent with the theoretical predictions of Equation 3. By minimizing the geometric factor “a”, micromotors maintain their mobility even in high‐salt environments (Figure S6 and Table S1).

To further validate the ability of 980 nm NIR light to penetrate tissue, we placed a 3 mm thick pork muscle in front of the NIR light and observed the micromotor's movement (see the schematic in Figure 4f and Movie S3). Even after penetrating the muscle tissue, the micromotors maintained directional motion in a PBS solution. As shown in Figure 4g, the mean square displacement (MSD) plot demonstrates that the micromotor maintains directional motion after tissue penetration. The inset of Figure 4g indicates a reduction in light intensity from 1.2 to 0.7 W cm^−2^ after passing through the muscle, which corresponded to a slight reduction in the micromotor's speed from 2.37 to 1.83 µm s^−1^. To further test their adaptability in complex biological environments, we evaluated MSWM motion within artificial blood vessels covered with a 3 mm‐thick muscle tissue in PBS solution (see the schematic in Figure 4h and Movie S4). As shown in Figure 4i, the MSWM demonstrates the ability to propel under 980 nm NIR light and navigate precisely through these artificial vessels with magnetic guidance.

Furthermore, to demonstrate the general applicability of ZIF‐8 coating for ion tolerance enhancement, we applied the coating to two other widely studied self‐diffusiophoresis micromotor systems: the SiO_2_/Pt‐based micromotor and the TiO₂/Pt Janus micromotor. Both systems exhibited similar improvements in ion tolerance (Figure S7 and Figure S8), validating the effectiveness of ZIF‐8 coatings in enhancing the performance of various MNMs designs.

### Cytotoxicity and pH‐Responsive Drug Release

**Figure 5:**
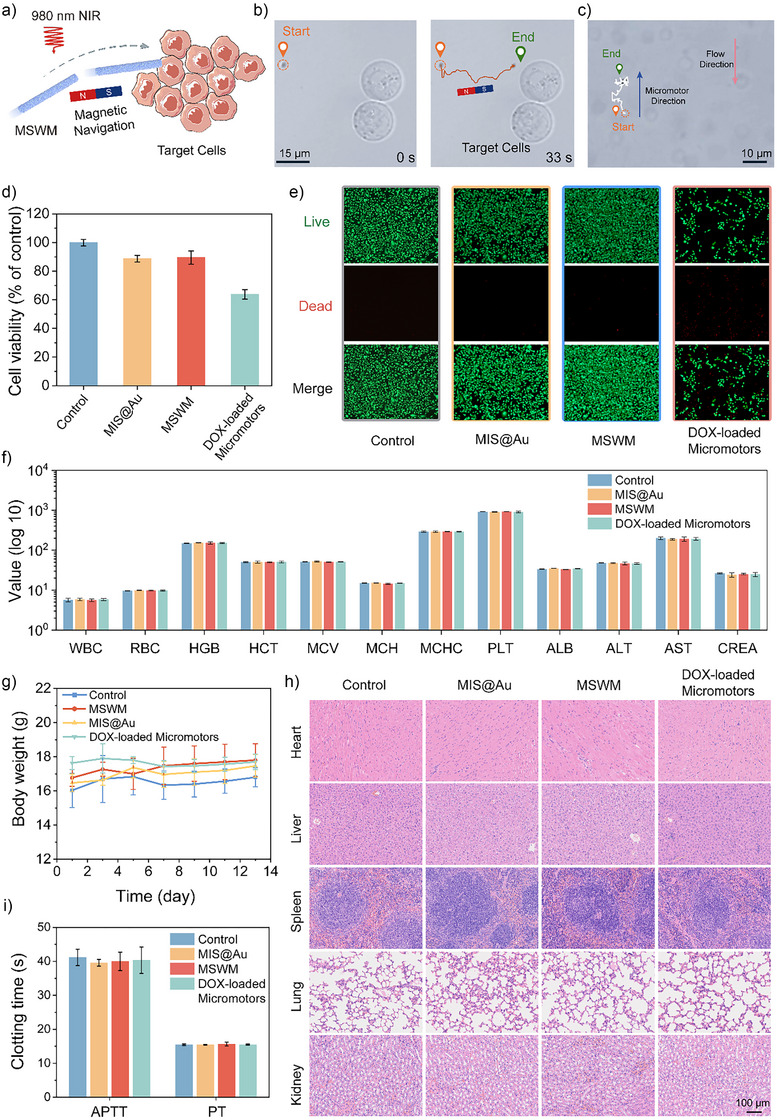
Assessment of MSWM in complex environments motion, biocompatibility, and anticoagulant properties. a) Schematic illustration of the MSWM manipulated toward the targeted cancer cells in the PBS solution. b) The trajectories of MSWM operation in PBS solution, together with the live EJ cells. c) The migration of MSWM in diluted blood. d) Viability of cancer cells (MCF‐7) after 24‐h incubation with MIS@Au, MSWM, DOX‐loaded Micromotors, and control groups. (*n* = 3; means ± SD). e) Fluorescence microscopy images of live (green) and dead (red) MCF‐7 cells after incubation with 32 µg mL^−1^ MIS@Au, 32 µg mL^−1^ MSWM, and 32 µg mL^−1^ DOX‐loaded micromotors. f) Routine hematological and blood biochemical tests of mice after 14 days of treatment. (*n* = 3; means ± SD). g) The body weight of mice before and after treatment in four experimental groups. (*n* = 3; means ± SD). h) H&E sections of major organs after treatment with different samples. i) In vitro anticoagulation activities of Control, MIS@Au, MSWM, and DOX‐loaded Micromotors. (*n* = 3; means ± SD).

In addition to enhancing ion tolerance, ZIF‐8 is highly biocompatible and offers significant drug‐loading capacity, making it a well‐suited nanocarrier for drug delivery applications.^[^ ^64^, ^65^, ^66^ ^]^ The porous structure of the ZIF‐8‐coated micromotors provides drug‐loading capacity as well as the pH‐responsive drug release capability. Coupled with the magnetic controllability of the silicon micromotors, this system enables precise targeted drug delivery (Figure 5a). As demonstrated in Figure 5b, the micromotors exhibited precise, controlled directional motion towards live cancer cells under 980 nm NIR light guided by a magnetic field (Movie S5).^[^ ^15^, ^67^, ^68^ ^]^ To further validate the motion performance of the MSWM in complex biological environments, we conducted experiments in a diluted blood medium. The results confirm that micromotors can successfully propel in flowing blood, demonstrating their potential applicability in dynamic biological environments (Figure 5c and Movie S6).

Imaging‐guided manipulation of MNMs is crucial for medical applications, and fluorescence imaging offers a simple and precise technique for real‐time tracking. Therefore, we investigated the fluorescence imaging capabilities of the MSWMs by loading the fluorescent dye RhB onto the ZIF‐8 crystals of MSWM, hereafter referred to as MSWM@RhB. This approach enables real‐time observation of micromotor movement. Figure S9 shows the fluorescent image of MSWM@RhB. Additionally, real‐time imaging of movement can be observed for MSWM@RhB under a fluorescence microscope (Figure S10 and Movie S7).

Figure S11 shows the quantification of Doxorubicin (DOX) concentrations using UV–Vis absorption spectroscopy, as detailed in the standard curve in Figure S12. Figure S13 shows the UV–Vis absorption spectroscopy for both the original DOX concentration of 60 µg mL^−1^ in the reaction solution and for the DOX concentration in the supernatant after reaction. The drug loading efficiency, calculated using the method described in the Experimental Section (Equations S6 and S7), was determined to be 45.06%. Moreover, MSWMs exhibit pH‐sensitive release profiles for DOX (Figure S14). In neutral or slightly alkaline conditions (pH 7.4), reflecting normal physiological environments, the ZIF‐8 structure remains stable, ensuring a slow, controlled release of DOX, aligning with targeted drug delivery goals. Conversely, in an acidic environment (pH 5.5) that simulates the tumor microenvironment, the ZIF‐8 framework undergoes degradation, which accelerates the release of DOX. This pH‐responsive behavior is crucial for targeted drug delivery, ensuring that higher concentrations of DOX are released at the acidic tumor site, thereby enhancing therapeutic efficacy while minimizing adverse effects on healthy tissues.

To be used for potential biomedical applications and to determine biocompatibility, micromotor should not be significantly cytotoxic.

To assess micromotor biodegradability, we examined the durability and degradation of the ZIF‐8 coating. SEM images (Figure S15) show that ZIF‐8 remains stable in PBS (pH 7.4) for 24 h, with gradual dissolution and minor structural collapse, whereas at pH 5.5, it degrades rapidly within 12 h. Furthermore, XRD patterns of the MIS@Au, original ZIF‐8, pristine MSWM, and MSWM after 24‐h immersion in PBS (pH 7.4) reveal that the characteristic crystalline peaks of ZIF‐8 are well‐preserved (Figure S16), supporting the conclusion that the MOF coating maintains good structural integrity in neutral physiological environments. Weight measurements further confirm minimal mass loss at pH 7.4 and rapid degradation at pH 5.5 (Figure S17). This pH‐responsive behavior ensures micromotor stability for targeted drug delivery while enabling controlled drug release in tumors, minimizing long‐term retention and biosafety risks. The degradation products, zinc ions and imidazole, are safely metabolized and excreted.

In addition to ZIF‐8 degradation, the biodegradability of the silicon‐based material used in micromotors is also crucial. Previous studies have demonstrated that silicon‐based materials can degrade in vivo,^[^ ^69^, ^70^ ^]^ confirming their degradation in physiological environments. This suggests that silicon‐based micromotors do not persist in the body, further mitigating biosafety concerns.

Additionally, we tested the cytotoxicity of the micromotor in fibroblasts. Figure S18 demonstrates that the MSWMs show inapparent cytotoxicity to normal cells after being cultured with fibroblasts for 24 h when the concentration was no more than 32 µg mL^−1^. Figure 5d compares the cytotoxic effectiveness of different micromotors against MCF‐7 cancer cells, revealing that the micromotor without DOX is non‐toxic, whereas the micromotor with DOX induces significant cytotoxicity in cancer cells. These live/dead staining images further corroborate the efficacy of the pH‐responsive system in selectively inducing cell death in cancerous conditions, thereby validating its potential as an effective cancer therapy. Figure 5e shows that green fluorescence marks live cells, while red fluorescence marks dead cells. Under acidic conditions, red fluorescence substantially increases, demonstrating the ability to kill cancer cells of the released DOX from the DOX‐loaded MSWMs.

Furthermore, we conducted biocompatibility and biosafety evaluations on mice over 14 days to confirm the safety of the micromotors for therapeutic applications. Blood routine tests include the indicators of white blood cells (WBC), red blood cells (RBC), hemoglobin (HGB), hematocrit (HCT), mean corpuscular volume (MCV), mean corpuscular hemoglobin (MCH), mean corpuscular hemoglobin concentration (MCHC), and platelets (PLT). Serum biochemical markers, including albumin (ALB), alanine aminotransferase (ALT), aspartate aminotransferase (AST), and creatinine (CREA), remained within normal ranges across all experimental groups (Figure 5f), indicating no systemic toxicity. Additionally, the stable weight gain of mice throughout the experiment (Figure 5g) further supports the excellent safety of the micromotors. H&E staining of major organs (heart, liver, spleen, lung, and kidney) (Figure 5h) showed no tissue damage or inflammatory response.

To evaluate the anticoagulation properties of the micromotors, clotting time assays were conducted in vitro. Specifically, the activated partial thromboplastin time (APTT) and prothrombin time (PT) were used as key indicators to assess anticoagulant effects. As shown in Figure 5i, the differences in clotting times between the sample groups and the control group were minimal, demonstrating that all tested samples exhibited favorable anticoagulant properties and posed no significant risk of blood coagulation.

These findings collectively demonstrate that the MSWMs exhibit high biocompatibility, controlled biodegradability, and minimal long‐term accumulation risks, making them a promising candidate for biomedical applications.

## Conclusion

In summary, this study introduces a novel strategy to enhance the ion tolerance of silicon‐based micromotors using a precise MOF coating (ZIF‐8) through a controllable heteroepitaxial growth process. The porous ZIF‐8 layer functions as ion‐conductive channels, maintaining the electrical “Debye layers” and preserving the self‐electrophoresis mechanism, even in high‐salt environments. By optimizing the ZIF‐8 coating and geometry factor, an *EI*₅₀ value of 5.322 ± 0.271 mM was achieved—improving ion tolerance by up to 266 times compared to uncoated micromotors. This enhancement allows stable motion in electrolyte concentrations up to 150 mM, comparable to blood salinity levels. Powered by 980 nm NIR light, these micromotors sustain propulsion even when covered by biological tissues due to NIR's deep tissue penetration. Additionally, the combination of magnetic properties and ZIF‐8′s porosity makes the micromotors ideal for targeted drug delivery. This MOF coating approach demonstrates versatility and scalability across other EMNMs, significantly enhancing their ion tolerance. By overcoming the ion tolerance limitations, this strategy paves the way for advanced biomedical applications, expanding the potential of EMNMs in future development.

## Conflict of Interests

The authors declare no conflict of interest.

## Supporting information



Supporting Information

Supporting Information

Supporting Information

Supporting Information

Supporting Information

Supporting Information

Supporting Information

Supporting Information

## Data Availability

The data that support the findings of this study are available in the Supporting Information of this article.
