# Supplementary material for: MOF Coating Enhances the Ion Tolerance of Micromotors
Source: Angew Chem Int Ed Engl. 2025 Jun 3;64(31):e202508001. doi: 10.1002/anie.202508001 (PMC12304825; doi:10.1002/anie.202508001)
Supplement: Supplementary file 1 — Supporting Information [file ANIE-64-e202508001-s002.docx]

*Supporting Information*

**MOF Coating Enhances the Ion Tolerance of Micromotors**

*Leyan Ou,^1^ Kunfeng Liu,^1^ Yifan Zhang,^3^ Wanyuan Li,^1^ Zixian Liang,^1^ Dapeng Lei,^1^ Hao Sun,^1^ Mojun Chen, ^4^ Jizhuang Wang,^1,^* Jinyao Tang,^2,^* Dan Li^1,^**

L. Ou, K. Liu, Y. Zhang, W. Li, Z. Liang, D. Lei, H. Sun, Dr. J. Wang, Prof. D. Li

^1^College of Chemistry and Materials Science, Jinan University, Guangzhou 510632, P. R. China; Guangdong Provincial Key Laboratory of Supramolecular Coordination Chemistry, Jinan University, Guangzhou, P. R. China.

Prof. J. Tang

^2^Department of Chemistry, The University of Hong Kong, Pokfulam Road, Hong Kong, P. R. China.

E-mail: jizhuang@jnu.edu.cn, jinyao@hku.hk, danli@jnu.edu.cn

Dr. Y. Zhang

^3^Guangdong Provincial Key Laboratory of Spine and Spinal Cord Reconstruction, The Fifth Affiliated Hospital (Heyuan Shenhe People's Hospital), Jinan University, Heyuan 517000, China.

Dr. M. Chen

^4^Smart Manufacturing Thrust, Systems Hub, The Hong Kong University of Science and Technology (Guangzhou), Guangzhou 511458, P. R. China.

Keywords: light-driven micromotor, NIR-driven, silicon micromotor, ion tolerance, MOF coating

**Table of Contents**

**Supplementary Methods**

1. Fabrication procedure of MSWM micromotor 7

2. Fabrication of MSWM@RhB 8

3. Micromotor migration measurement procedure and data analysis 8

4. PEC photocurrent measurement of MSWM micromotor 9

5. Characterization methods 9

6. pH-responsive drug release 9

7. Biocompatibility and cytotoxicity in vitro 10

8. Cell Imaging 10

9. Degradation rate of ZIF-8 in different pH conditions 11

10. Biocompatibility in vivo 11

**Supplementary Discussion**

1. Numerical simulation for ion distribution and electric field 12

2. Calculation of IPCE 13

3. The simulation of the geometry factor "𝑎" 13

4. The calibration of DOX concentration 14

5. The calculation of drug loading efficiency 14

**Supplementary Figures**

**Figure S1**. Results of the experimental and simulated (model) SE spectra (psi and delta) of SiO_2_–Si.

**Figure S2**. Line-scan across the Ni-deposited interface on the Si wafer.

**Figure S3**. The hysteresis loop of the MSWM microwires separated from the substrate. The inset is an enlarged part.

**Figure S4**. Zeta potential of Si microwires, MIS microwires, MIS@Au microwires, ZIF-8, and MSWM microwires. Error bars represent the SD of the averaged values from three measurements.

**Figure S5.** Scanning Electron Microscopy (SEM) image of the MSWMs array on the silicon substrate with different lengths.

**Figure S6**. (a-p) The representative ionic strength dependence of the normalized speed for MSWMs with various geometric factors "*a*". The solid lines represent the predicted theoretical relationship. In the inset, the solid lines and corresponding color bands indicate the fitting plots with the confidence interval of the experimental data at 95% confidence level. The dashed lines indicate the expected theoretical relationship. Error bars represent the SD of the averaged values from three measurements.

**Figure S7**. (a) Scanning Electron Microscopy (SEM) image of the SiO_2_-Pt @ZIF-8 micromotor (b) SiO_2_-Pt micromotor in 70 μM ferrocenemethanol (MFc) without (black) and with (red) ZIF-8 coating. Error bars represent the SD of the averaged values from three measurements.

**Figure S8**. (a) Scanning Electron Microscopy (SEM) image of the TiO_2_-Pt @ZIF-8 micromotor. (b) TiO_2_-Pt micromotor in 70 μM ferrocenemethanol (MFc) without (black) and with (red) ZIF-8 coating. Error bars represent the SD of the averaged values from three measurements.

**Figure S9**. (a) Fluorescence image of MSWM@RhB. (b) Non-fluorescence image of MSWM@RhB.

**Figure S10**. The trajectory of a MSWM@RhB.

**Figure S11.** UV-Vis absorption spectra for various DOX concentration.

**Figure S12**. The standard curve of DOX concentration (μg/mL) versus absorption intensity. The absorption intensity of different concentrations of DOX solutions prepared by dissolving DOX in DI water measured by UV-Vis method.

**Figure S13.** UV-Vis spectra of DOX before and after loading onto MSWMs.

**Figure S14.** DOX release profiles from MSWMs under different pH conditions.

**Figure S15.** Images of ZIF-8 coated silicon-based micromotor in different PBS environments and various immersion times.

**Figure S16.** XRDs of MIS@Au, origin ZIF-8, origin MSWM and MSWM after immersion at PBS pH=7.4 during 24 h.

**Figure S17.** Degradation rate of ZIF-8 in different PBS environments and various immersion times.

**Figure S18**. The viability of fibroblast cell with Si, MIS@Au and MSWM after incubation for 24 h. Error bars represent the SD of the averaged values from three measurements.

**Supplementary Tables**

**Table S1**. Geometric factor "*a*" and *EI_50_* of ZIF-8 coated micromotor.

**Supplementary Movies**

**Movie S1:** Migration comparison of the micromotors with and without ZlF-8 coating under a chopped light.

**Movie S2:** The migration of a typical MSWM in NaCl solutions of different ionic strengths.

**Movie S3:** The migration of a MSWM in PBS solution under the 980 nm NIR irradiation, without (orange line) and with (blue line) the irradiation passing through a 3 mm layer of pork muscle tissue.

**Movie S4:** Migration of the micromotor in artificial vessel under magnetic navigation.

**Movie S5:** Migration of the micromotor guided around the cells under magnetic navigation in PBS solution.

**Movie S6:** The migration of MWSM in diluted blood.

**Movie S7:** The migration of a typical MSWM@RhB under fluorescence microscope.

**Experimental Methods**

1. **Fabrication procedure of MSWM micromotor**

The substrate used was a n-type <100> silicon wafer (supplied by Suzhou Hengli New Materials Co., Ltd.). The first step involved sequential ultrasonic cleaning of the silicon wafer in deionized water, acetone, and isopropanol, each for 5 minutes, followed by nitrogen drying. Subsequently, the silicon wafer was immersed in a Piranha solution (composed of 98% H_2_SO_4_ and 50% H_2_O_2_ in a 5:1 v/v ratio) for 15 minutes to remove organic residues from the silicon surface. After thorough rinsing with deionized water, the silicon wafer was further cleaned by soaking in a buffered HF (BHF) solution with a 5:1 ratio. Following BHF etching, the silicon wafer was rinsed with deionized water, resulting in the formation of a series of pores on the SiO_2_ layer. These pores had a diameter of approximately 2 μm with a spacing of 4 μm. The patterning was achieved via photolithography using AZ-5214 photoresist, followed by metal-assisted chemical etching. The silicon wafer was then immersed in a metal-assisted chemical etching solution composed of 5 M HF and 0.02 M AgNO_3_ for 80 minutes to promote the growth of silicon microwires. Afterward, the microwires were soaked in acetone for 5 minutes to remove the AZ-5214 photoresist and then rinsed with deionized water. To further clean the silicon microwires, they were soaked in HNO_3_ for 20 minutes to remove any residual silver on the surface. The microwires were then thermally oxidized at 1000°C in an oxygen environment to grow a 100 nm thick SiO_2_ layer over 30 minutes, followed by removal of the SiO_2_ layer using a 5:1 buffered HF (BHF) solution. The microwires were subjected to oxygen plasma treatment for 120 seconds, resulting in a SiO_2_ insulating layer. Finally, a 25 nm nickel (Ni) layer was deposited onto the silicon microwires using thermal evaporation on a custom tilt-rotating platform. To optimize electrochemical reactions, gold nanoparticles were deposited using magnetron sputtering. and subsequently dried to obtain the MIS@Au micromotors.

The prepared MIS@Au micromotors were subjected to oxygen plasma treatment for hydroxyl functionalization. Subsequently, Precursor solutions were prepared by dissolving 4.105 g of 2-methylimidazole in 50 ml of deionized water. Simultaneously, 0.183 g of Zn(CH_3_COO)_2_·2H_2_O was dissolved in 10 ml of deionized water to prepare the zinc ion solution. The previously prepared MIS@Au micromotor was immersed in the 2-methylimidazole solution for 3 minutes. Subsequently, the zinc precursor solution was added to the 2-methylimidazole solution. The reaction was carried out at room temperature and atmospheric pressure for 30, 40, 50, and 60 minutes. After the reaction, the silicon wafer was thoroughly washed with deionized water and methanol, and subsequently dried to obtain the MSWMs.

1. **Fabrication of MSWM@RhB**

The fabrication of MSWM@RhB micromotors was performed as previously described.^[1]^ RhB (5 mg) was dissolved in an aqueous solution containing Zn(CH_3_COO)_2_·2H_2_O. The MIS@Au micromotor array on the silicon wafer was immersed in an aqueous solution of 2-methylimidazole for 3 minutes, after which the RhB-containing metal solution was added to the mixture and allowed to react at room temperature for 50 minutes. Following the reaction, the silicon wafer was thoroughly washed with deionized water and methanol, and subsequently dried to obtain the MSWM@RhB micromotors.

1. **Micromotor migration measurement procedure and data analysis**

The silicon microwire micromotors were carefully scraped off the substrate using a blade and dispersed into a 70 µM ferrocenemethanol (MFc) solution to measure their migration behavior. The conductivity of the solution was adjusted by adding a high concentration of NaCl to the 70 µM MFc solution. Measurements were taken once the conductivity meter readings stabilized. Video recordings were conducted using a brightfield optical microscope (Olympus MX51), a near-infrared laser emitter, and a digital camera. Video recordings were executed using a 50X objective lens at a resolution of 1920 × 1080 pixels and a frame rate of 25 frames per second. The migration velocity was analyzed using a custom MATLAB program. The center of mass of each individual frame indicates the position of the microrobot within that frame. The motion trajectory of the micromotor is obtained by calculating each frame in turn.

1. **PEC photocurrent measurement of MSWM micromotor**

The photocurrent of the MSWM micromotor in the MFc solution was evaluated using a three-electrode setup. In this setup, n-Si served as the cathode, MIS as the anode, and Ag/AgCl as the reference electrode. The electrodes were immersed in a solution containing 70 μM MFc and 1 M NaCl. Measurements were conducted using a PXI-4132 Precision Source Measurement Unit (National Instruments) under short-circuit conditions, with the current measured at zero bias.

1. **Characterization methods**

Scanning Electron Microscope (SEM) images was captured using a 15 kV field emission SEM (S4700). Energy dispersive X-ray (EDX) mapping was captured by Bruker 6160. Transmission Electron Microscope (TEM) images were acquired it a Talos F200S G2 operating at 120 kV. Atomic Force Microscope (AFM) images were obtained using a Bruker Bioscope Catalyst. Magnetic properties of the samples were tested by Lake-Shore vibration sample magnetometer (VSM) with a sensitivity of 10^−7^ emu.

1. **pH-responsive drug release**

The sample of drug-loaded micromotors (MSWM@DOX) was dispersed in 5 mL of buffer solution of pH=7.4 (Gibco) and pH=5.5 (Ecotop), and the drug release was conducted at 37°C with constant shaking. At regular time intervals, the samples were centrifuged at 8000 rpm for 5 minutes, and 1 mL of the supernatant from each sample were collected and analyzed using UV-Vis spectrophotometry at 480 nm. Subsequently, 1 mL of fresh PBS solution was added to the release system to continue the drug release process. The amount of DOX released from MSWM@DOX at each time point was determined by measuring the UV-Vis absorption spectrum of the supernatant and fitting it to a standard calibration curve. This data was used to calculate the cumulative release of DOX from MSWM@DOX over time.

1. **Biocompatibility and cytotoxicity in vitro**

To evaluate the biocompatibility of MSWM, fibroblasts were used. To assess the cytotoxicity of MSWM, MCF-7 cells (Human Breast Carcinoma Cells) were used.

To evaluate the biocompatibility of MSWM, the fresh culture medium containing various concentrations of MSWM (0, 2, 4, 8, 16, 32,64 μg/mL) was prepared and used to replace the original medium in 24-well plates(1×10^4^ cells per well). Each concentration was tested in triplicate. After incubating the cells for 24 hours, they were washed with phosphate-buffered saline (PBS, pH 7.4; Gibco). Subsequently, CCK-8 working solution (Targetmol) was added to the 24-well plates, and the plates were incubated in the dark for 2 h. The absorbance of each well was measured at 450 nm using a microplate reader (Epoch, BIOTEK, USA).

To assess the cytotoxicity of MSWM, fresh culture medium with the same composition was used to evaluate the cytotoxicity of MIS@Au (32 μg/mL), MSWM (32 μg/mL), and DOX-loaded micromotors (32 μg/mL). These were also added to 24-well plates (1×10^4^ cells per well). After culturing for 24 h, the CCK-8 solution was added to the 24-well plates, followed by another 2-hour incubation in the dark. The absorbance was then measured using a microplate reader.

1. **Cell Imaging**

The viability of MCF-7 cells was assessed using a Live-Dead Cell staining kit (Acridine Orange/Ethidium Bromide (AO/EB) assay kit, Solarbio). After a pre-treatment time, the cells were washed three times with PBS. Subsequently, the cells were incubated in AO/EB solution at room temperature for 10 minutes, followed by PBS wash. The cells were then immediately observed under a fluorescence microscope (Vert.A1, Zeiss Axio). The control group consisted of cells without the addition of materials.

**9.** **Degradation rate of ZIF-8 in different pH conditions**

To evaluate the degradation behavior of ZIF-8 under different pH conditions, ZIF-8 was dispersed in phosphate-buffered saline (PBS) at pH 7.4 and pH 5.5 at a concentration of 0.5 mg/mL.^[2]^ At predetermined time intervals (30 min, 1h, 3h, 6h, 12h, and 24h), the remaining ZIF-8 was collected and weighed to determine its degradation rate.

**10.** **Biocompatibility in vivo**

We performed routine hematological and biochemical analyses on BALB/C mice after 14 days of treatment. The mice were randomly divided into four groups (n=3) and intravenously injected with PBS, MIS@Au, MSWM, MSWM@DOX every two days. Body weight was monitored throughout the experiment. On Day 14, blood samples were collected via orbital venipuncture. Additionally, blood routine and biochemical tests were conducted using blood samples collected from the eyeballs of experimental mice.

Wound specimens from mice on Day 14 were fixed in 4% formaldehyde, embedded in paraffin, and sectioned into 4 μm thick slices for histological analysis. The tissue sections were then stained with hematoxylin and eosin (H&E) for microscopic observation.

All procedures were performed in accordance with the National Institutes of Health Guidelines for the Care and Use of Laboratory Animals and approved by the Ethics Committee at Jinan University.

**Supplementary Discussion**

1. **Numerical simulation for ion distribution and electric field**

# The distribution of charged ions and fluid velocity was simulated using the COMSOL Multiphysics software. We adapted 2D models as we previously reported. ^[3]^ With MFc as the reversible redox, ferrocenium cations are generated at the Ni (anode) surface and reduced at the n-Si (cathode) surface. The distribution of charged ions follows Equation S1, which plays a key role in establishing an electric field and propelling the micromotor's motion:

$\nabla j_{i}=u\cdot\nabla c_{i}-D_{i}\nabla^{2}c_{i}-\frac{z_{i}FD_{i}\nabla\cdot(c_{i}\nabla\emptyset)}{RT}$ S1

where $j_{i}$ is the flux of ion *i* and the three terms on the right of the equation represent the contribution of convection, diffusion, and migration, respectively. In addition, $u$ is the fluid velocity, $\emptyset$is the electrostatic potential, *R* is the gas constant, *F* is the Faraday constant, *T* is the temperature and $c_{i}$ is the concentration of ion *i*, $D_{i}$ is the diffusion coefficient of ion *i*, $z_{i}$ is the charge of ion *i*.

The generation of the electric field *E* (*E*= -∇*φ*) resulting from the presence of unbalanced ions is calculated using the Poisson equation:

$-\varepsilon_{0}\varepsilon_{r}\nabla^{2}\emptyset=\rho_{e}=F_{Z_{+}C_{+}}$ S2

Where *φ* is the electric potential, $\varepsilon_{0}$ is the vacuum permittivity and $\varepsilon_{r}$ is the relative electric permittivity of water, $Z_{+}=+1$, $\rho_{e}$is the volumetric charge density, $F$ is the Faraday constant and $C_{+}$ is the concentration of the corresponding cations, respectively.


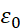

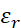


The boundary condition was established using the zeta potentials of the MIS@Au micromotor (-16.4 mV) and the MSWMs (26.1 mV). Steady ionic fluxes at both the anode and cathode surfaces were derived from the measured photocurrent density.

1. **Calculation of IPCE**

$IPCE=\frac{\left| j_{p}-j_{d} \right|\times1240}{\lambda\times P_{in}}\times100\%$ S3

Where $j_{p}$ is the photocurrent density (mA·cm^-2^), $j_{d}$ is the dark current density (mA·cm^-2^), $\lambda$ is the wavelength of incident monochromatic light (nm), $P_{in}$ is the Light power density on the photoelectrode (mW·cm^-2^).

1. **The simulation of the geometry factor "𝑎"**

It is a dimensionless ratio of the surface conductivity 𝐾^𝜎^ to the fluid bulk conductivity 𝐾^𝐿^ multiplied by the characteristic length of the particle "𝑎". In the case of our microswimmer, the geometry factor "𝑎" can be defined as:

$\alpha=\frac{L\beta}{4\pi r}$ S4

$R_{solution}=\frac{1}{\beta K^{L}}$ S5

where 𝑅_𝑠𝑜𝑙𝑢𝑡𝑖𝑜𝑛_ is the effective resistance from anode to cathode through the solution. A 3D core-shell model was built by the commercial COMSOL Multiphysics package as we previously reported. 𝑅_𝑠𝑜𝑙𝑢𝑡𝑖𝑜𝑛_ under different solution conductivity and different geometry can be calculated by the electric current module through applying a certain voltage between the two electrodes.

1. **The calibration of DOX concentration**

The standard curve can be expressed according to the Bier-Lambert law as follows:

$A=0.03759+0.03328 C$ S6

where $A$ is the absorbance intensity of UV-Vis spectrums, $C$ is the concentration of DOX. The correlation coefficient (R^2^) is 0.9999.

1. **The calculation of drug loading efficiency**

The drug loading efficiency can be calculated using the following formula:

$Drug loading efficiency \left( \% \right)= (C_{0}-C_{S})/C_{0} \times100\%$ S7

where $C_{0}$ is the initial concertation of DOX in the reaction solution, $C_{S}$ is the concertation of DOX in the supernatant after reaction.

**Supplementary Figures**

**
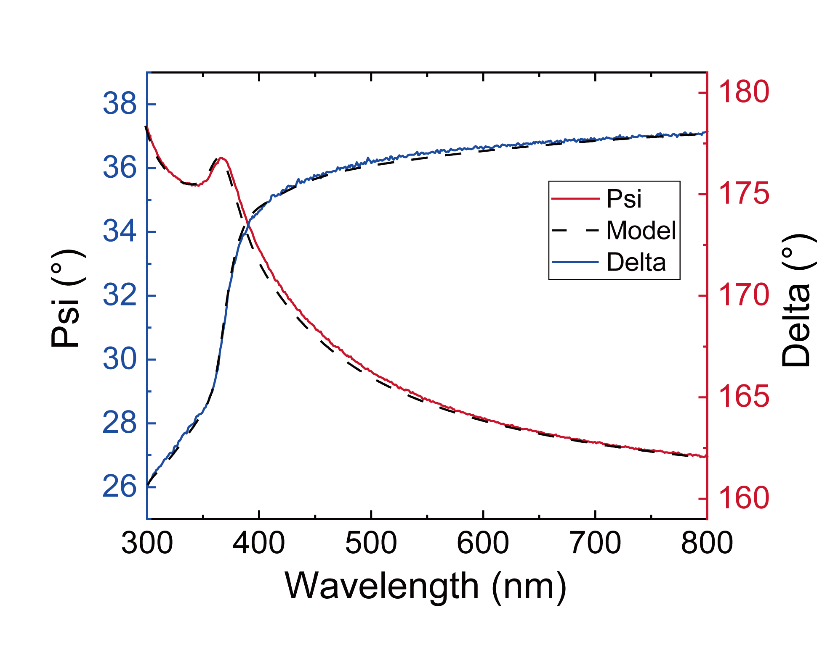
**

**Figure S1.** Results of the experimental and simulated (model) SE spectra (psi and delta) of SiO_2_–Si.

**
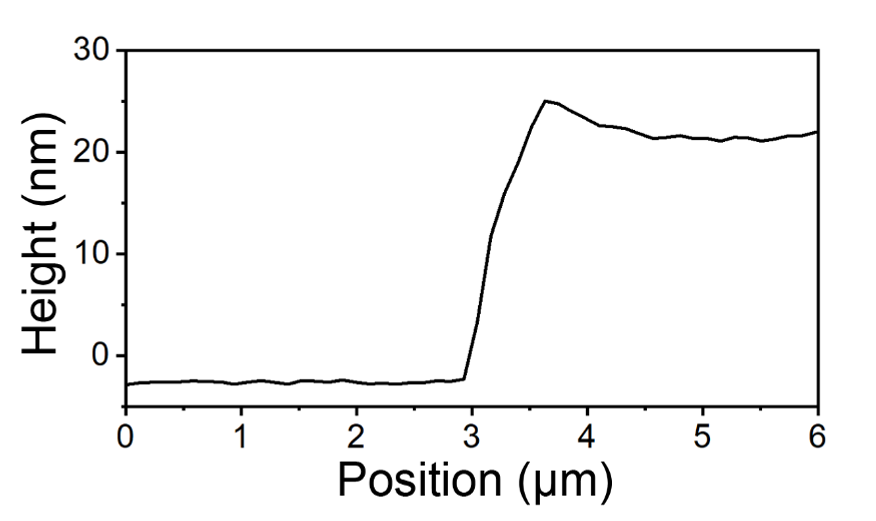
**

**Figure S2.** Line-scan across the Ni-deposited interface on the Si wafer.


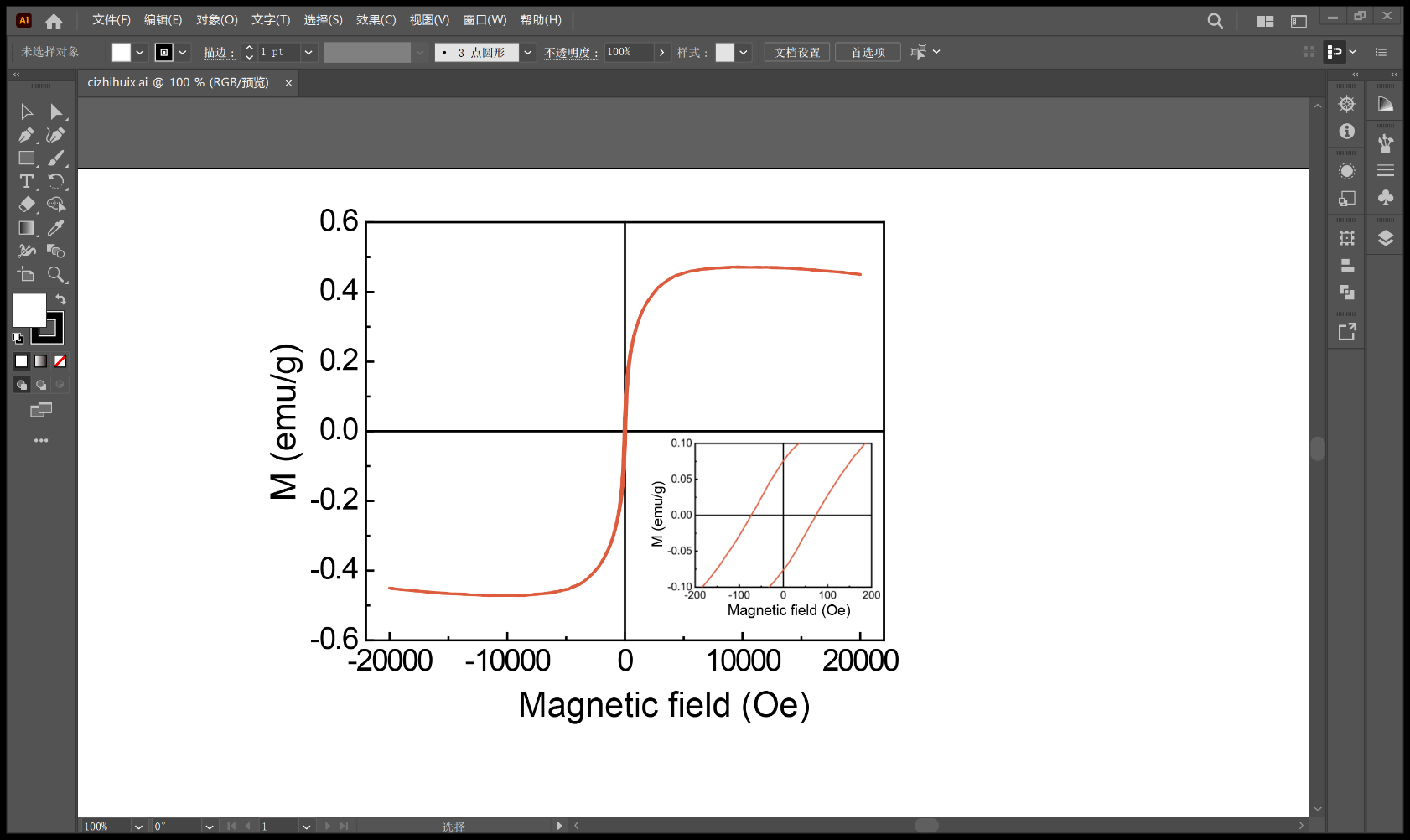


**Figure S3.** The hysteresis loop of the MSWM microwires separated from the substrate. The inset is an enlarged part.

**
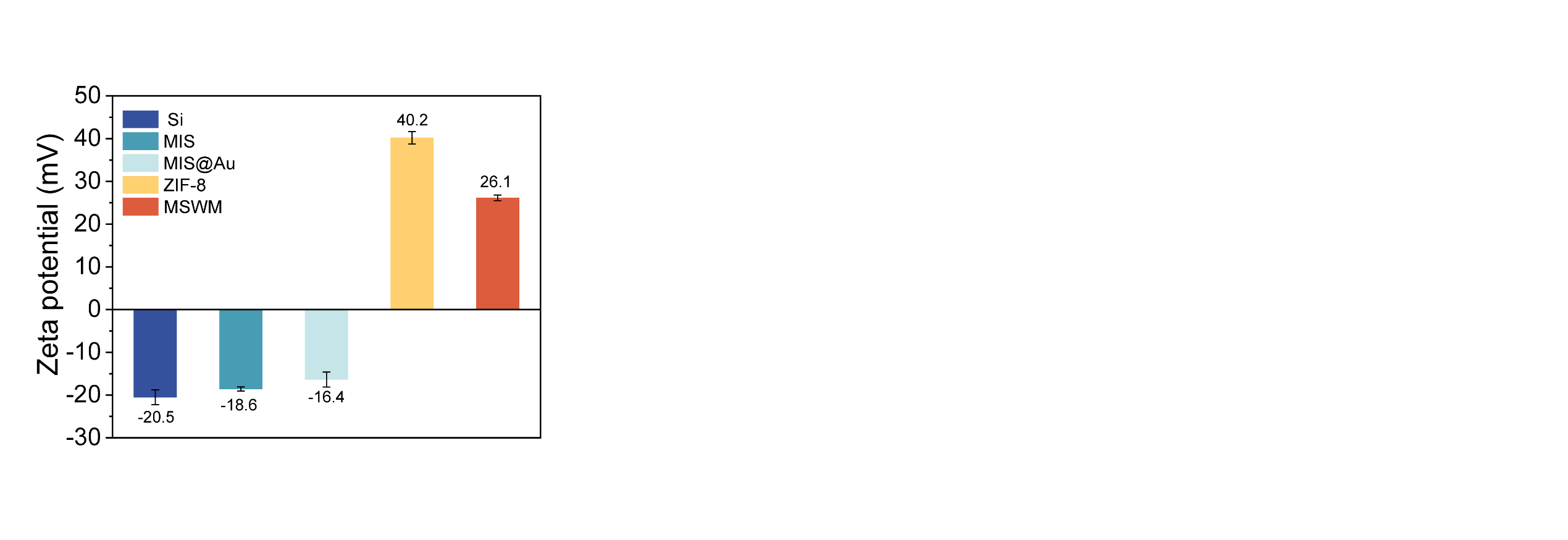
**

**Figure S4.** Zeta potential of Si microwires, MIS microwires, MIS@Au microwires, ZIF-8, and MSWM microwires. Error bars represent the SD of the averaged values from three measurements.

**
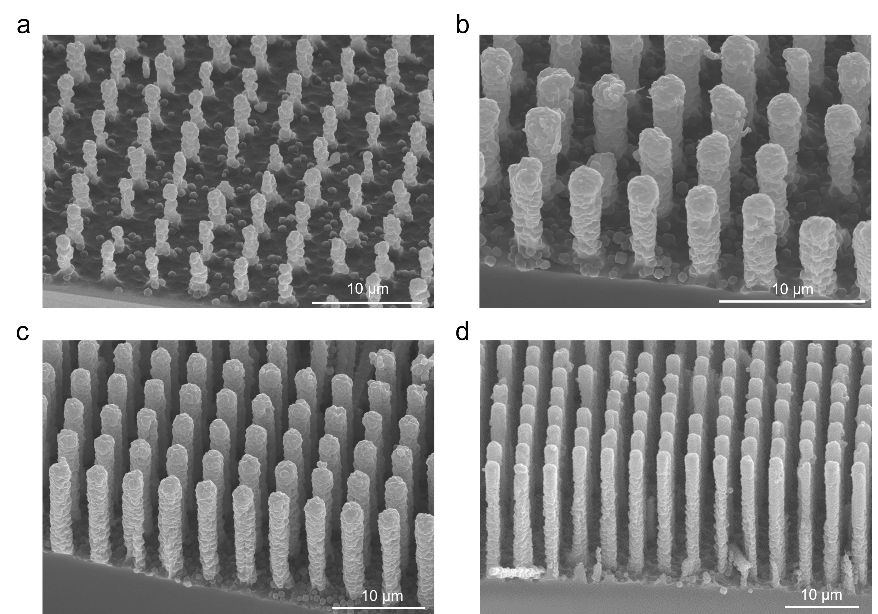
**

**Figure S5.** Scanning Electron Microscopy (SEM) image of the MSWMs array on the silicon substrate with different lengths.

**
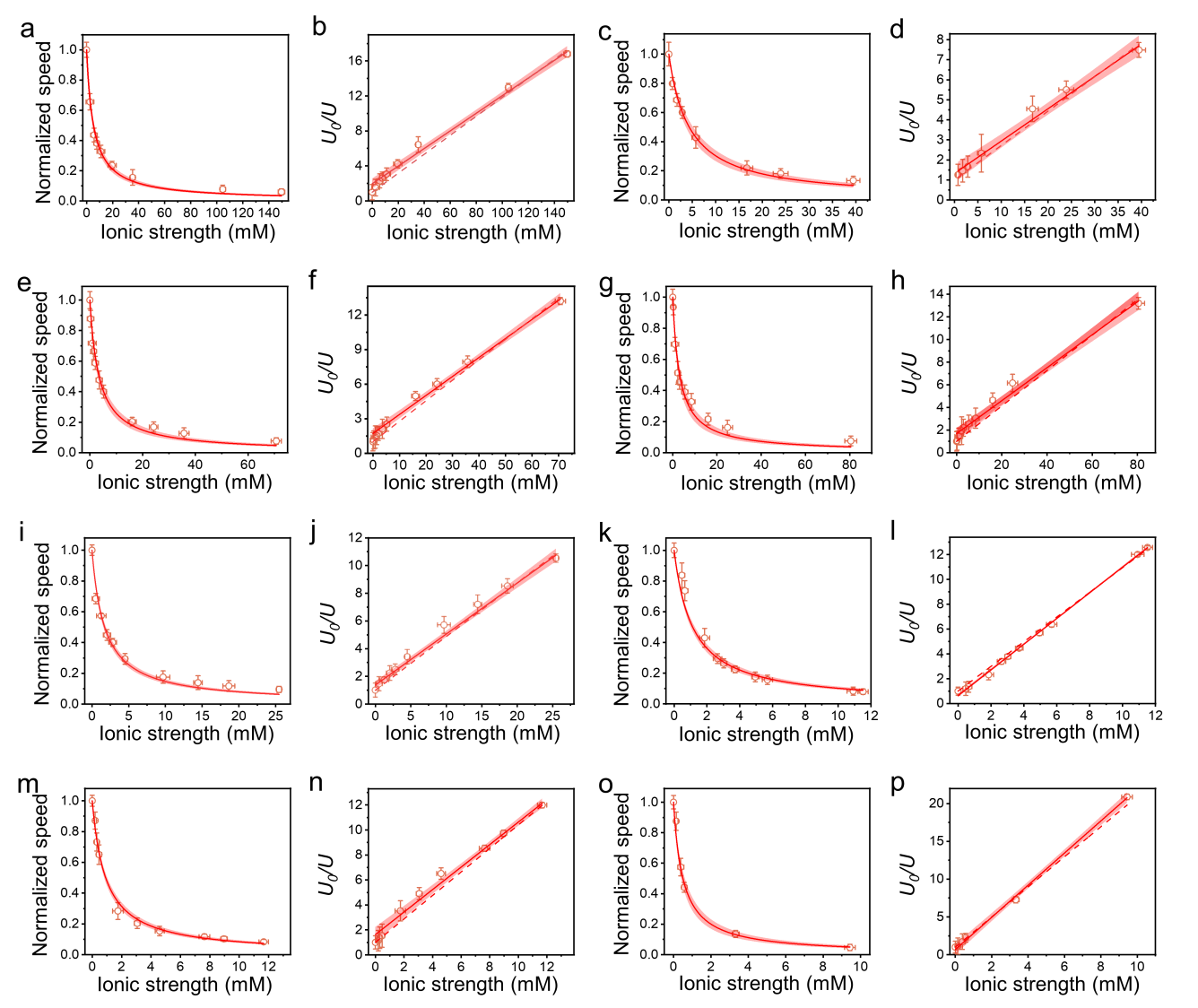
**

**Figure S6.** (a-p) The representative ionic strength dependence of the normalized speed for MSWMs with various geometric factors "*a*". The solid lines represent the predicted theoretical relationship. In the inset, the solid lines and corresponding color bands indicate the fitting plots with the confidence interval of the experimental data at 95% confidence level. The dashed lines indicate the expected theoretical relationship. Error bars represent the SD of the averaged values from three measurements.

**
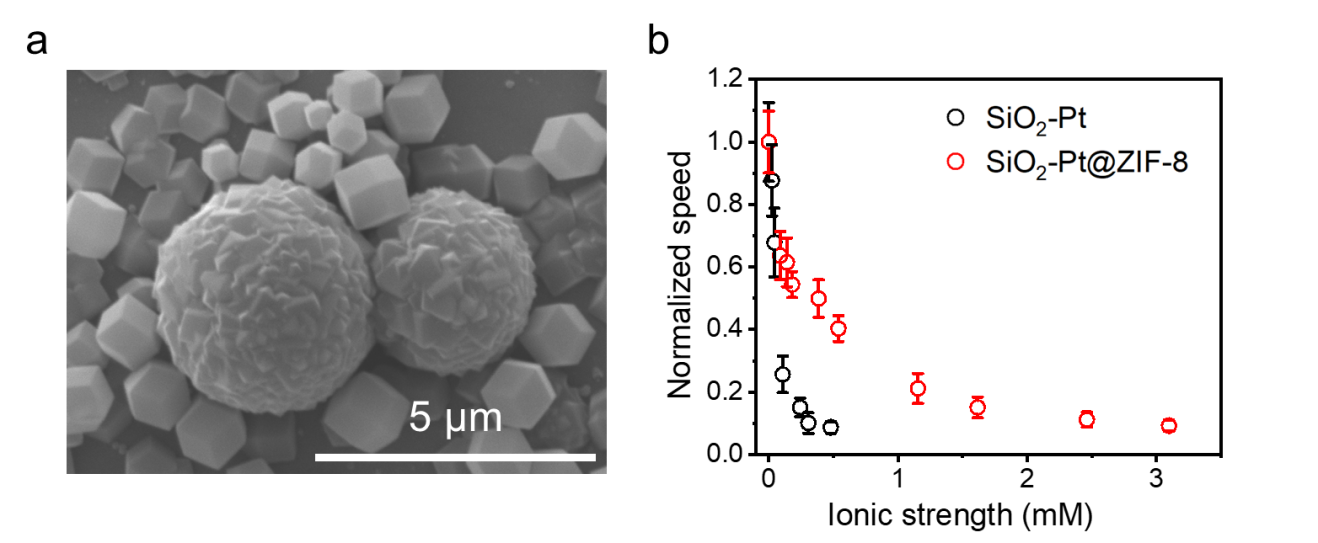
**

**Figure S7.** (a) Scanning Electron Microscopy (SEM) image of the SiO_2_-Pt@ZIF-8 micromotor (b) SiO_2_-Pt micromotor in 70 μM ferrocenemethanol (MFc) without (black) and with (red) ZIF-8 coating. Error bars represent the SD of the averaged values from three measurements.

**
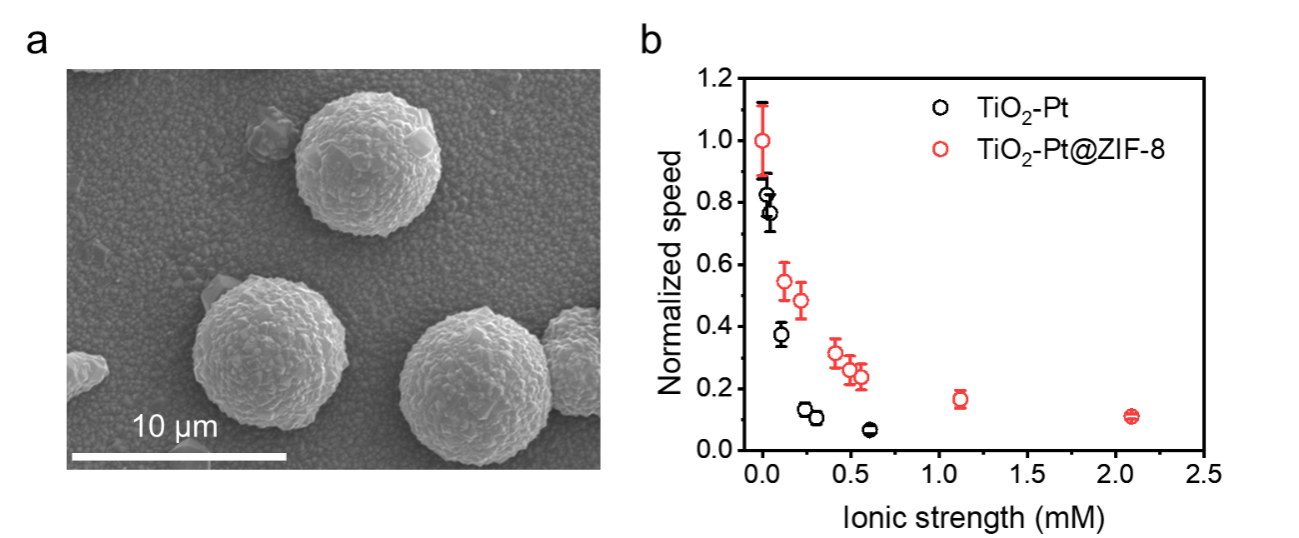
**

**Figure S8.** (a) Scanning Electron Microscopy (SEM) image of the TiO_2_-Pt@ZIF-8 micromotor. (b) TiO_2_-Pt micromotor in 70 μM ferrocenemethanol (MFc) without (black) and with (red) ZIF-8 coating. Error bars represent the SD of the averaged values from three measurements.


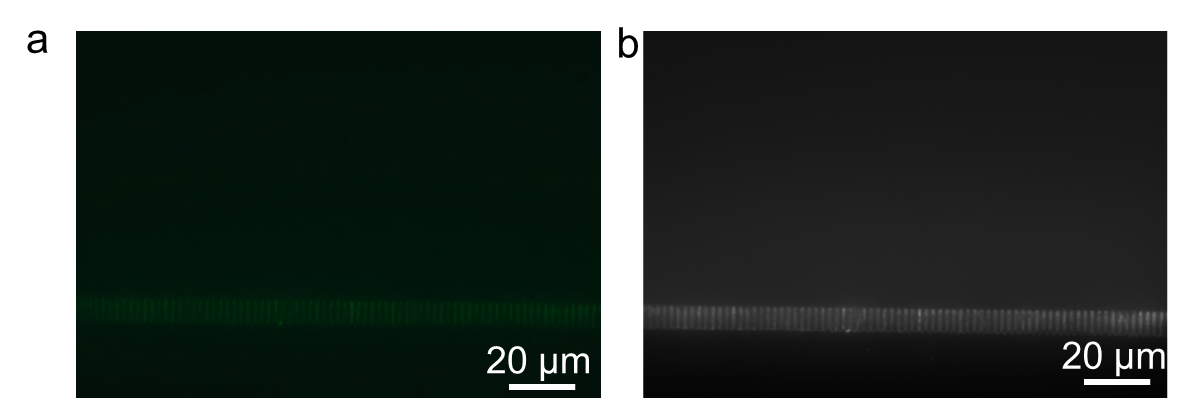


**Figure S9.** (a) Fluorescence image of MSWM@RhB. (b) Non-fluorescence image of MSWM@RhB.

**
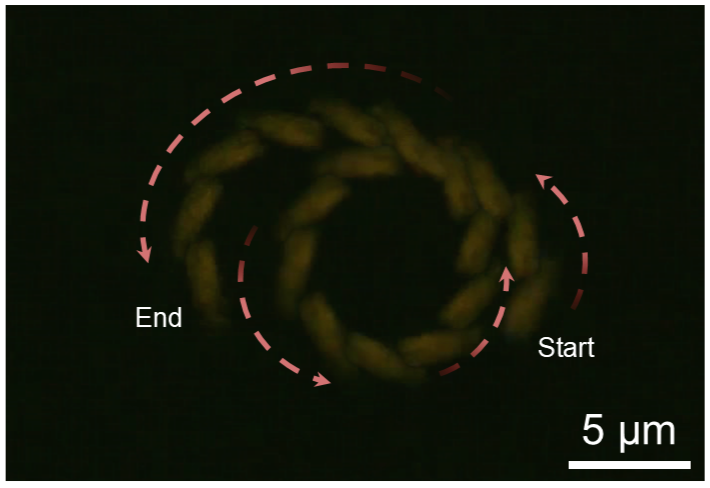
**

20 μm

**Figure S10.** The trajectory of a MSWM@RhB.

**
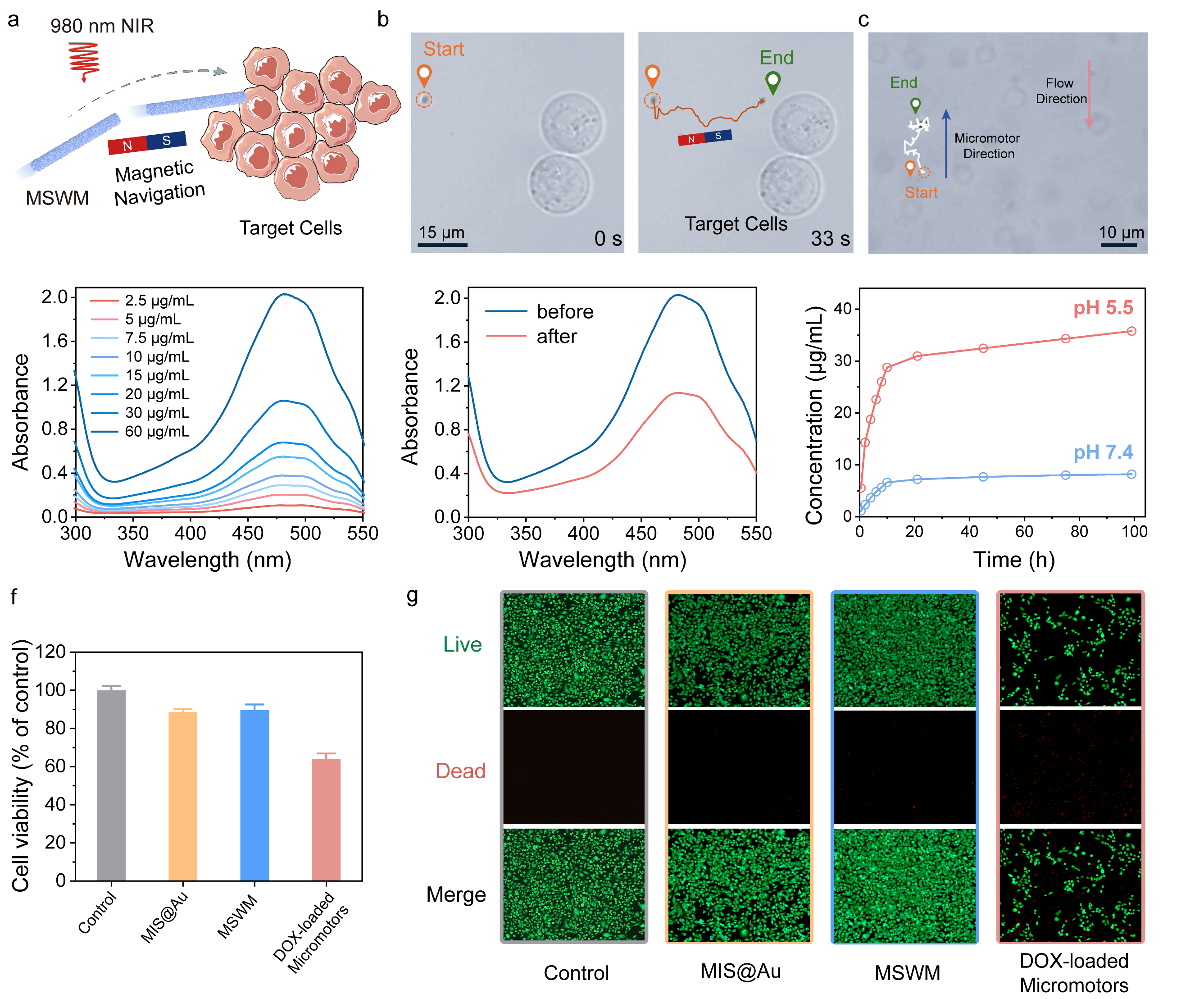
**

**Figure S11.** UV-Vis absorption spectra for various DOX concentration.

**
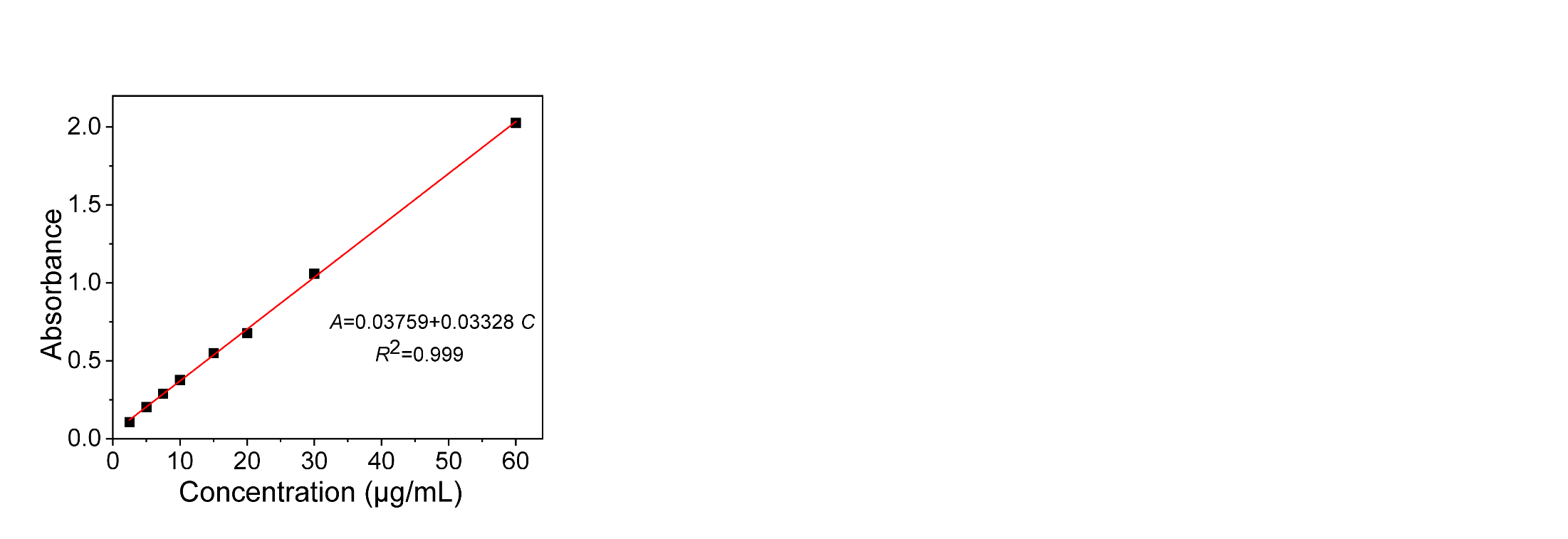
**

**Figure S12.** The standard curve of DOX concentration (μg/mL) versus absorption intensity. The absorption intensity of different concentrations of DOX solutions prepared by dissolving DOX in DI water measured by UV-Vis method.


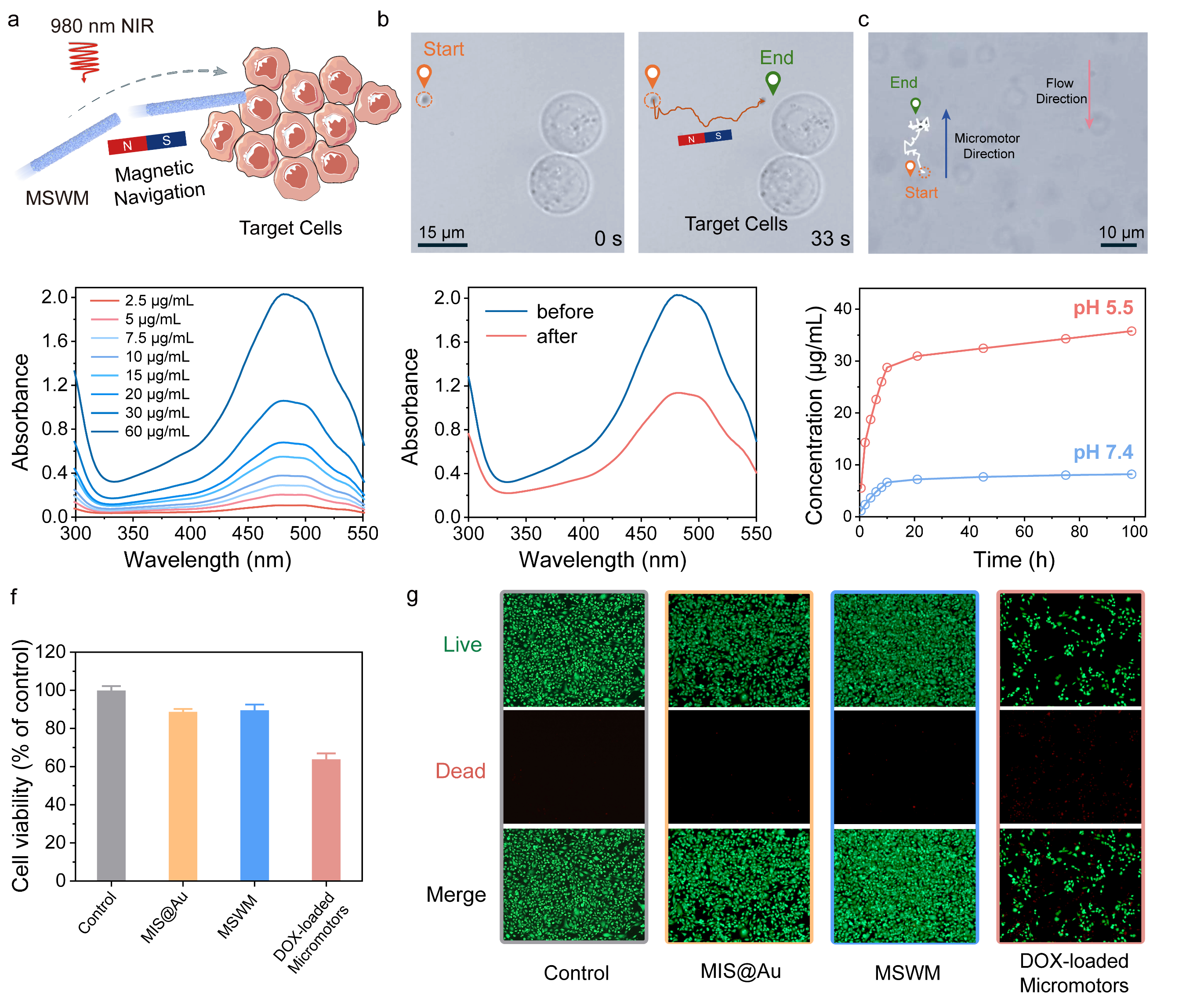


**Figure S13.** UV-Vis spectra of DOX before and after loading onto MSWMs.


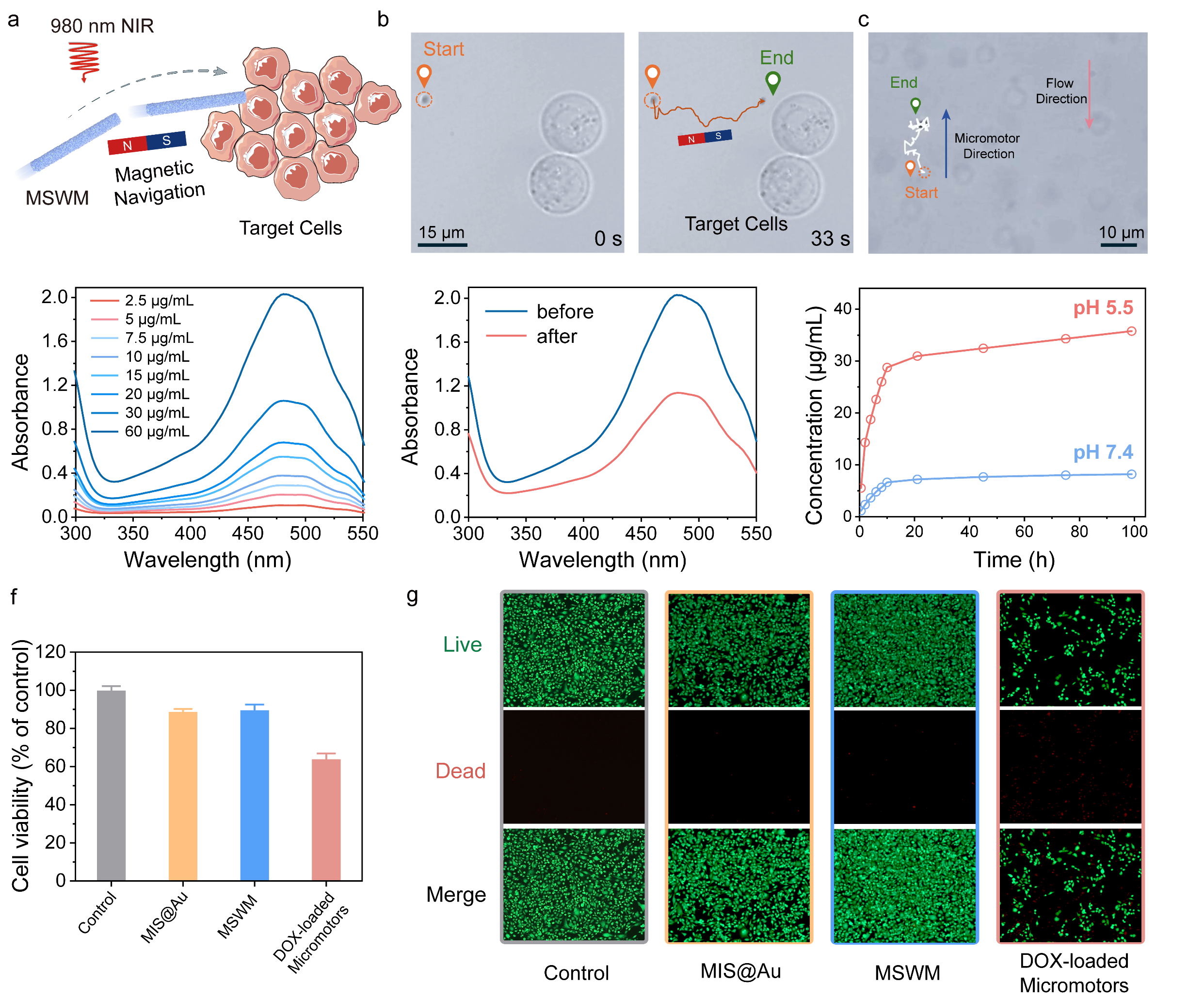


**Figure S14.** DOX release profiles from MSWMs under different pH conditions.


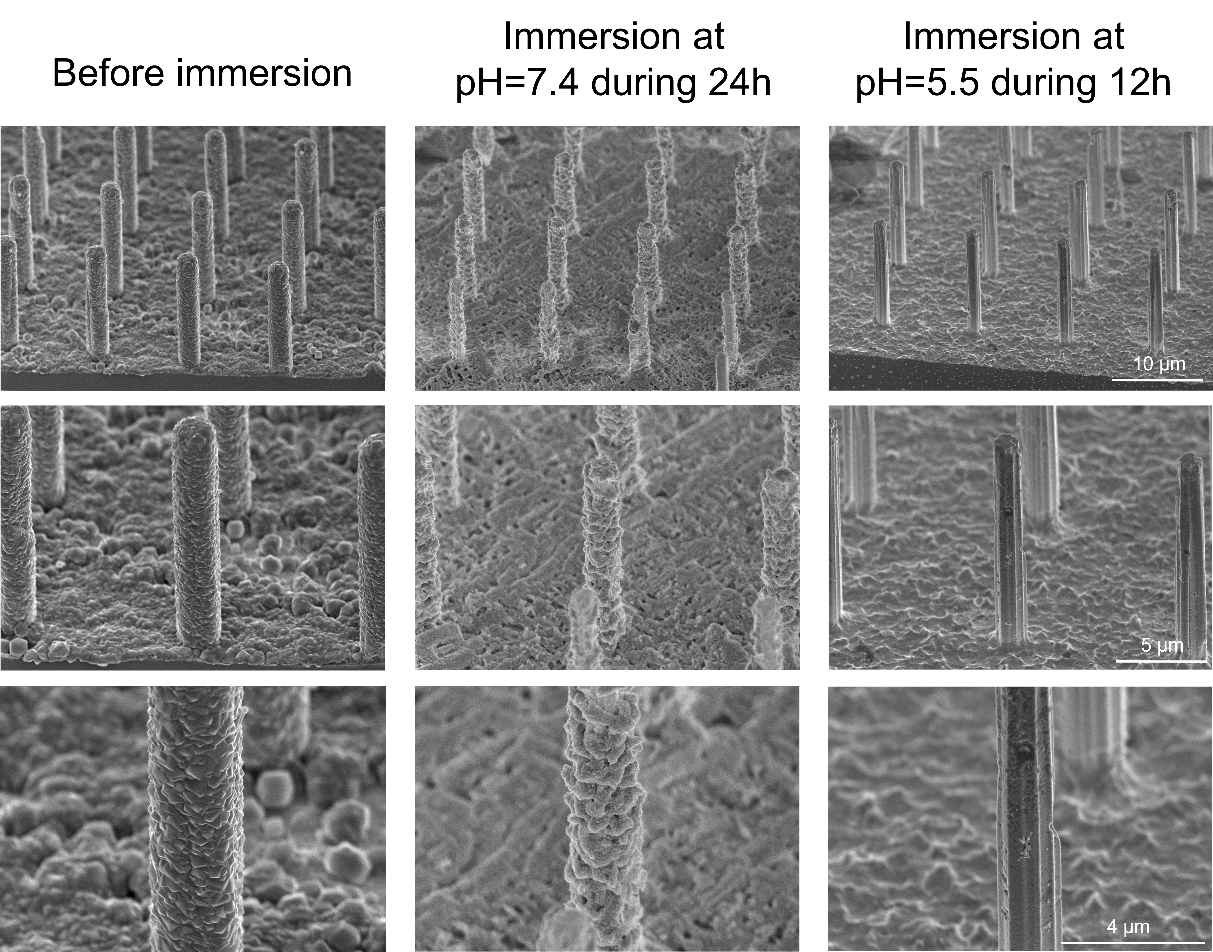


**Figure S15.** Images of ZIF-8 coated silicon-based micromotor in different PBS environments and various immersion times.


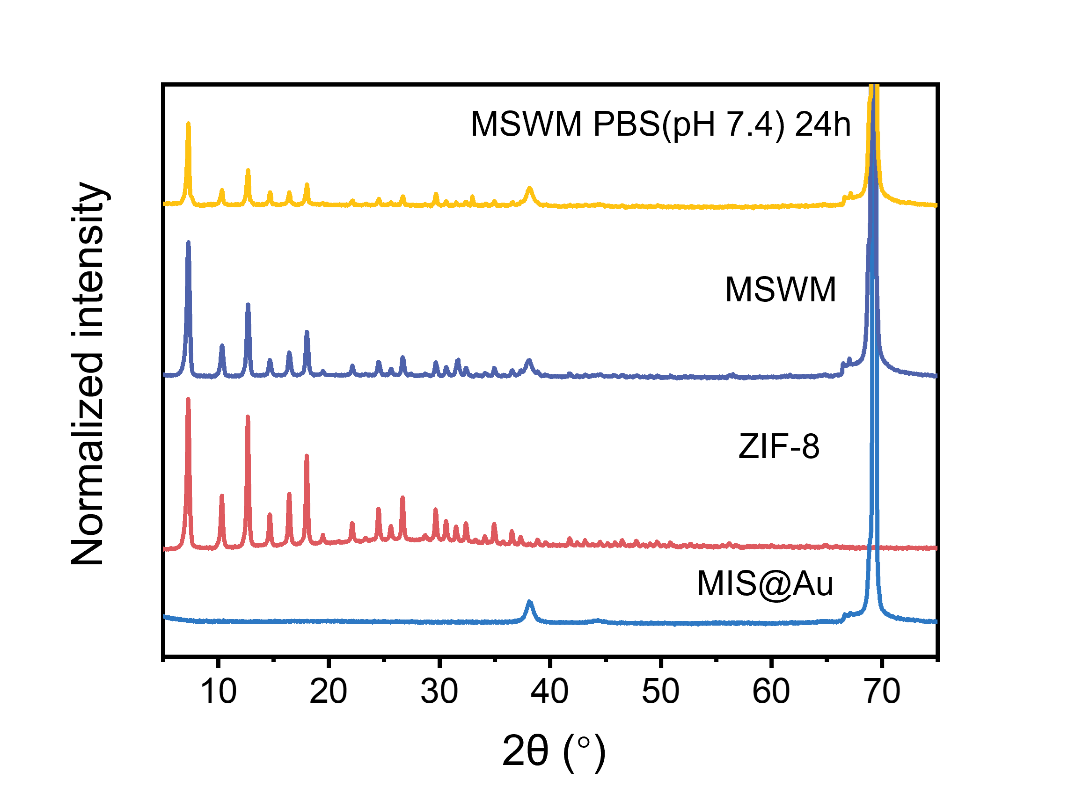


**Figure S16.** XRDs of MIS@Au, origin ZIF-8, origin MSWM and MSWM after immersion at PBS pH=7.4 during 24 h.

**
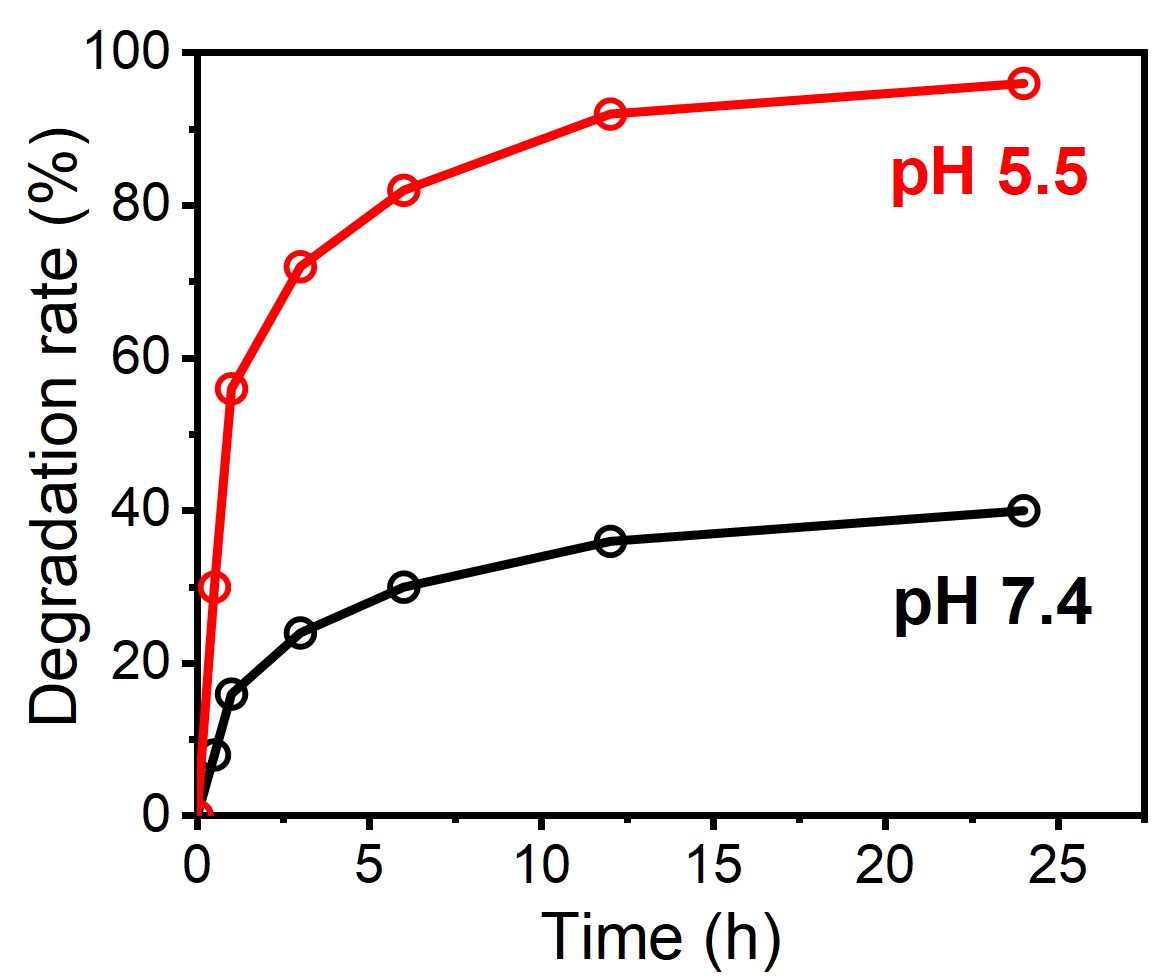
**

**Figure S17.** Degradation rate of ZIF-8 in different PBS environments and various immersion times.

**
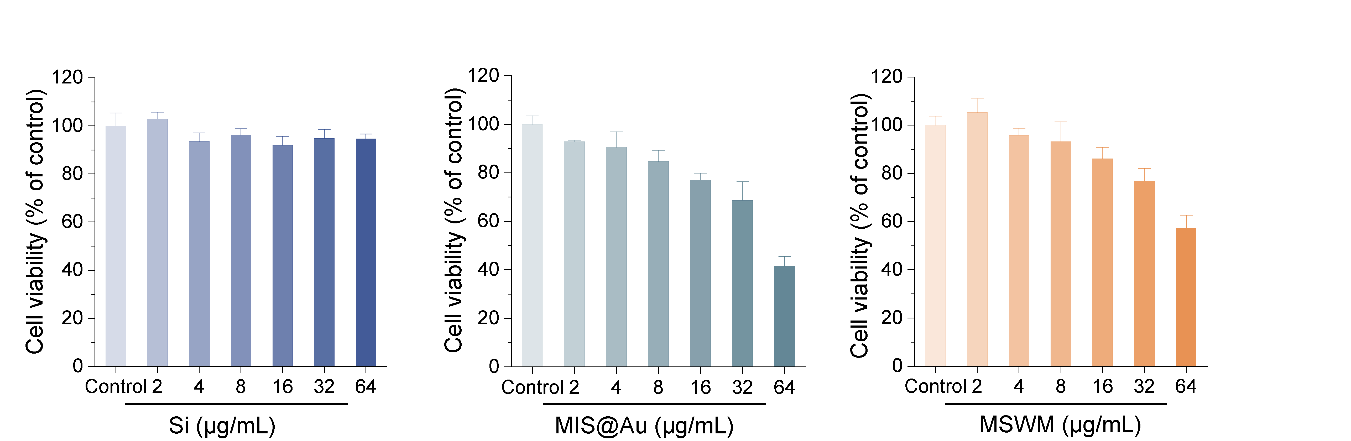
**

**Figure S18**. The viability of fibroblast cell with Si, MIS@Au and MSWM after incubation for 24 h. Error bars represent the SD of the averaged values from three measurements.

**Supplementary Table**

**Table S1.** Geometric factor "*a*" and *EI_50_* of ZIF-8 coated micromotor.

| **ZIF-8 Coated Micromotor** | **Geometric factor "*a*" (μm)** | ***EI_50_* (mM)** |
| --- | --- | --- |
| **a** | **0.87** | **5.322±0.271** |
| **c** | **1.23** | **4.369±0.268** |
| **e** | **1.46** | **3.500±0.233** |
| **g** | **1.58** | **3.146±0.253** |
| **i** | **3.04** | **1.724±0.069** |
| **k** | **5.35** | **1.135±0.047** |
| **m** | **6.23** | **0.917±0.039** |
| **o** | **12.62** | **0.504±0.031** |

#

# **References**

[1] X. Wang, X.-Z. Chen, C. C. J. Alcântara, S. Sevim, M. Hoop, A. Terzopoulou, C. de Marco, C. Hu, A. J. de Mello, P. Falcaro, S. Furukawa, B. J. Nelson, J. Puigmartí-Luis, S. Pané, *Adv. Mater.* **2019**, *31*, 1901592.

[2] M. d. J. Velásquez-Hernández, R. Ricco, F. Carraro, F. T. Limpoco, M. Linares-Moreau, E. Leitner, H. Wiltsche, J. Rattenberger, H. Schröttner, P. Frühwirt, E. M. Stadler, G. Gescheidt, H. Amenitsch, C. J. Doonan, P. Falcaro, *CrystEngComm* **2019**, *21*, 4538-4544.

[3] a) J. Wang, Z. Xiong, X. Zhan, B. Dai, J. Zheng, J. Liu, J. Tang, *Adv. Mater.* **2017**, *29*, 1701451; b) J. Wang, Z. Xiong, M. Liu, X.-m. Li, J. Zheng, X. Zhan, W. Ding, J. Chen, X. Li, X. D. Li, S.-P. Feng, J. Tang, *ACS Nano* **2020**, *14*, 3272-3280.
